# Outcomes of Bypass Surgery in Adult Moyamoya Disease by Onset Type

**DOI:** 10.1001/jamanetworkopen.2024.15102

**Published:** 2024-06-06

**Authors:** Yong Cheol Lim, Eunyoung Lee, Jihye Song

**Affiliations:** 1 Department of Neurosurgery, Ajou University School of Medicine, Ajou University Hospital, Suwon, Republic of Korea; 2 Department of Neurology, McGovern Medical School, The University of Texas Health Science Center at Houston

## Abstract

**Importance:**

Moyamoya disease (MMD) is a rare chronic cerebrovascular disease, and the outcomes of bypass management in adult patients remain controversial.

**Objective:**

To categorize adult MMD based on asymptomatic, ischemic, and hemorrhagic onset and compare the outcomes (death, hemorrhagic stroke [HS], and ischemic stroke [IS]) of bypass surgery (direct or indirect) with those of conservative management.

**Design, Setting, and Participants:**

This retrospective, nationwide, population-based longitudinal cohort study used Korean National Health Insurance Research data to identify adults (aged ≥15 years) with MMD who were diagnosed between January 1, 2008, and December 31, 2020, and followed up until December 31, 2021 (median follow-up, 5.74 [IQR, 2.95-9.42] years). A total of 19 700 participants (3194 with hemorrhagic, 517 with ischemic, and 15 989 with asymptomatic MMD) were included. Data were analyzed from January 2 to April 1, 2023.

**Exposures:**

Bypass surgery and conservative management.

**Main Outcomes and Measures:**

Death constituted the primary outcome; secondary outcomes consisted of HS or IS. Kaplan-Meier survival curve and Cox proportional hazards regression analysis were applied. The propensity score–matching and stratified analyses were performed to control covariate effects.

**Results:**

A total of 19 700 patients (mean [SD] age, 45.43 [14.98] years; 12 766 [64.8%] female) were included. Compared with conservative management, bypass was associated with a reduced risk of death (adjusted hazard ratio [AHR], 0.50 [95% CI, 0.41-0.61]; *P* < .001) and HS (AHR, 0.36 [0.30-0.40]; *P* < .001) in hemorrhagic MMD; reduced risk of IS (AHR, 0.55 [95% CI, 0.37-0.81]; *P* = .002) in ischemic MMD; and reduced risk of death (AHR, 0.74 [95% CI, 0.66-0.84]; *P* < .001) in asymptomatic MMD. However, bypass was associated with an increased risk of HS (AHR, 1.76 [95% CI, 1.56-2.00]; *P* < .001) in asymptomatic MMD. Both direct and indirect bypass demonstrated similar effects in hemorrhagic and asymptomatic MMD, except only direct bypass was associated with a reduced risk of IS (AHR, 0.52 [95% CI, 0.33- 0.83]; *P* = .01) in ischemic MMD. After stratification, bypass was associated with a reduced risk of death in patients younger than 55 years with ischemic (AHR, 0.34 [95% CI, 0.13- 0.88]; *P* = .03) and asymptomatic (AHR, 0.69 [95% CI, 0.60-0.79]; *P* < .001) MMD, but an increased risk of HS in patients 55 years or older with ischemic MMD (AHR, 2.13 [95% CI, 1.1-4.16]; *P* = .03).

**Conclusions and Relevance:**

The findings of this cohort study of bypass outcomes for patients with MMD emphasize the importance of tailoring management strategies in adult patients based on onset types.

## Key Points

**Question:**

What are the outcomes of bypass surgery in patients with adult moyamoya disease (MMD) with varying onset types?

**Findings:**

In this population-based cohort study of 19 700 patients, bypass surgery was associated with reduced risk of death and hemorrhagic stroke in hemorrhagic MMD, ischemic stroke in ischemic MMD, and death in asymptomatic MMD; however, bypass was associated with an increased risk of hemorrhagic stroke in asymptomatic MMD. Both direct and indirect bypass showed similar outcomes in asymptomatic and hemorrhagic MMD, except that only direct bypass was associated with a reduced risk of ischemic stroke in ischemic MMD.

**Meaning:**

These findings suggest that it may be beneficial to tailor management strategies for patients with adult MMD based on onset type.

## Teaser

This cohort study categorizes patients with adult moyamoya disease based on asymptomatic, ischemic, and hemorrhagic onset and compares bypass surgery and conservative management in terms of the risk of death, hemorrhagic stroke, and ischemic stroke.

## Introduction

Moyamoya disease (MMD) is a rare cause of stroke characterized by progressive stenosis of the terminal portion of the internal carotid arteries and compensatory capillary collaterals.^1,2,3^ The prevalence of MMD is estimated to be 0.1 per 100 000 population worldwide but is higher in East Asian countries, especially Korea (6.3 to 16.1 per 100 000) and Japan (3.16 to 10.5 per 100 000).^4,5,6,7^ Moyamoya disease presents with a bimodal age distribution, at approximately 10 years and 30 to 50 years.^1,2^ Bypass surgery usually improves prognosis in pediatric patients. However, the benefits and the optimal modality of bypass in adult patients remain controversial. Randomized clinical trials and large studies suggesting the superiority of a specific management modality have been scarce. They are limited by the small size or heterogeneity (eg, children or adults, presenting symptoms, and varying disease progression status on angiographic findings) of the cohorts and present outcomes without considering their onset types.

To address these limitations, we used the Korean Health Insurance Review and Assessment (HIRA) database, which has a special registration program for rare intractable diseases (RID), including MMD. We categorized the patients based on onset type (asymptomatic, ischemic, and hemorrhagic MMD) and compared the effect of bypass surgery (direct or indirect) with conservative management in adult patients with MMD in terms of the risk for death, hemorrhagic stroke (HS), and ischemic stroke (IS). This approach aims to provide a more comprehensive understanding of the optimal management for adult patients with MMD and guide clinicians in making tailored treatment decisions.

## Methods

### Study Participants, Comorbidity, and Outcome Variables

This retrospective, population-based, longitudinal cohort study used the RID program in the HIRA claims database based on the Korean population.^8,9,10^ The study was approved by the Ajou University Hospital Institutional Review Board. Access to the HIRA database was approved by the Korea National Health Insurance Sharing Service. Informed consent was waived owing to the use of deidentified data. The study followed the Strengthening the Reporting of Observational Studies in Epidemiology (STROBE) reporting guideline.

**Figure 1.**
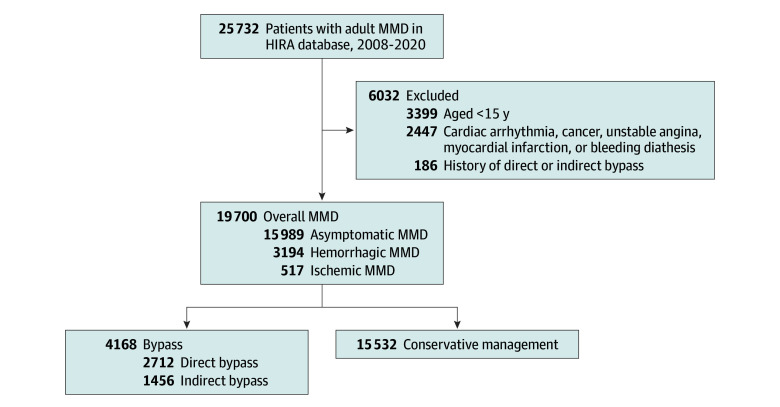
Flowchart of the Study HIRA indicates Korean Health Insurance Review and Assessment database; MMD, moyamoya disease.

We identified patients newly diagnosed with MMD from January 1, 2008, to December 31, 2020, and we followed them up to December 31, 2021. A 1-year washout period (2007-2008) was used to prevent prevalent cases from interfering with the data for inclusion, exclusion, and confounding factors.^11,12^ We improved the accuracy of patient identification by using specific operational definitions. The diagnosis of MMD was established if *International Statistical Classification of Diseases, Tenth Revision* (*ICD-10*), code I67.5 and RID code V128 were newly recorded at least once in the database at hospital discharge or more than twice in the outpatient department. Patients with bilateral and unilateral involvement were both included in our data analysis.^10^ The exclusion criteria were as follows: (1) younger than 15 years^11^; (2) a history of direct or indirect bypass surgery before a diagnosis of MMD; (3) cardiac arrhythmia (*ICD-10* code I48) that may cause thromboembolic complications; (4) cancers (*ICD-10* codes C); (5) unstable angina or myocardial infarction (*ICD-10* codes I20-I25) within the past 12 months; and (6) bleeding diathesis (*ICD-10* codes D65-D69). We classified the identified patients into 3 groups based on the following onset types: hemorrhagic, ischemic, and asymptomatic MMD. We categorized the patients according to the following management strategies: direct bypass, indirect bypass, and conservative management (Figure 1).

The primary outcome was the occurrence of death; secondary outcomes were HS or IS based on *ICD-10* codes. Baseline comorbidities were defined as conditions diagnosed within 1 year before the MMD diagnosis date: hypertension, type 2 diabetes, and dyslipidemia^13,14,15,16^ (eDiscussion in Supplement 1). Detailed information about covariates and outcomes are described in the eMethods in Supplement 1.

### Statistical Analysis

Data were analyzed from January 2 to April 1, 2023. Kaplan-Meier survival curves were generated and compared using the log-rank test. The proportional hazard assumption was tested by Schoenfeld residuals, and Cox proportional hazards regression models were adopted to calculate hazard ratios (HRs) and 95% CIs. A 1:1 propensity score–matching analysis and stratified analyses were performed to control for confounding factors. Detailed statistical methods are described in the eMethods in Supplement 1. Any missing data were excluded from the analysis. All statistical analyses were 2 sided, and *P* < .05 indicated statistical significance. All analyses were performed using SAS Enterprise Guide, version 6.3 (SAS Institute Inc). All plots were drawn with R, version 4.1.2 (R Project for Statistical Computing).

## Results

### Baseline Characteristics

Of the 25 732 patients with newly diagnosed MMD, 6032 were excluded. A total of 19 700 patients (mean [SD] age, 45.43 [14.98] years; 12 766 [64.8%] female and 6934 [35.2%] male) were followed up for 124 819 person-years from diagnosis. The median follow-up was 5.74 (IQR, 2.95-9.42) years. The baseline characteristics according to onset type and MMD management are summarized in eTables 1 and 2 in Supplement 1. The number of bypass surgical procedures in all MMD and hemorrhagic MMD were examined (eTable 3 in Supplement 1). The proportion of direct surgical procedures increased (*P* for trend, <.001) and a notable change was observed in all MMD (coefficient, −0.078; Wald χ^2^ = 4.06; *P* = .04) and the hemorrhagic MMD (coefficient, −0.608; Wald χ^2^ = 8.30; *P* = .004) when comparing before and after 2014. For bypass vs conservative management, fewer deaths and HS were observed in hemorrhagic MMD and fewer IS in ischemic MMD. Increased HS was observed in asymptomatic MMD comparing direct and indirect bypass, but only direct bypass showed reduced IS in ischemic MMD.

**Figure 2.**
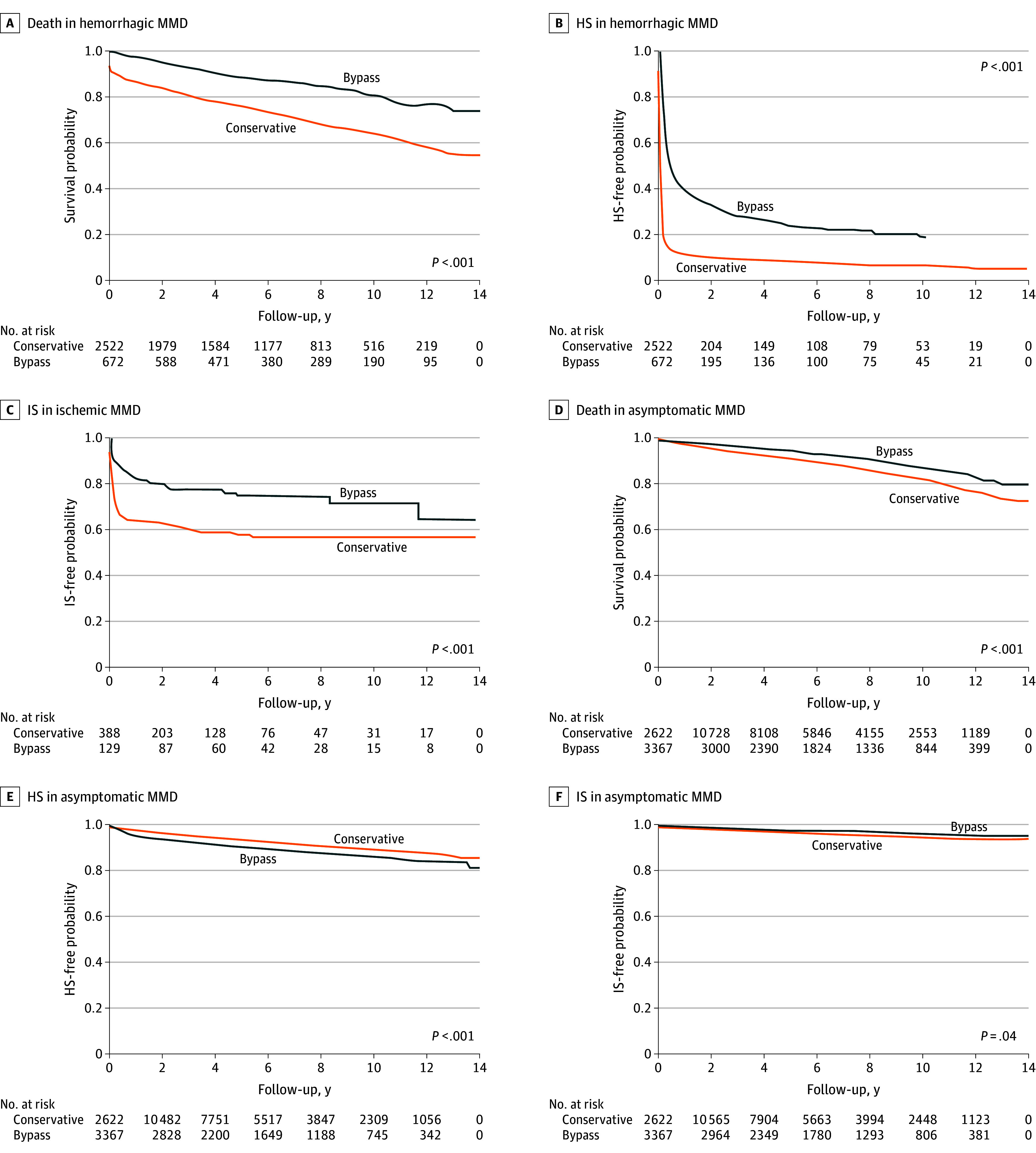
Patient Outcomes After Bypass Surgery HS indicates hemorrhagic stroke; IS, ischemic stroke; MMD, moyamoya disease.

Kaplan-Meier curves were used to compare the risks of death, HS, and IS between the bypass and conservative management groups (Figure 2 and eFigure 1 in Supplement 1) and among the direct and indirect bypass and conservative management groups (eFigure 2 in Supplement 1). Compared with conservative management, bypass was associated with a reduced risk of death in all MMD (*P* < .001) (eFigure 1 in Supplement 1); a reduced risk of death and HS in hemorrhagic MMD (*P* < .001) (Figure 2); a reduced risk of IS in ischemic MMD (*P* < .001); and a reduced risk of death (*P* < .001) and IS (*P* = .04) in asymptomatic MMD. Notably, bypass was associated with increased risk of HS in asymptomatic MMD (*P* < .001). Among direct bypass, indirect bypass, and conservative management (eFigure 2 in Supplement 1), direct or indirect bypass was associated with reduced risk of death (*P* < .001), HS (*P* = .007), and IS (*P* = .04) in all MMD, and a similar association was found in other subgroups except IS in asymptomatic MMD (*P* = .10).

Cox proportional hazards regression analysis was performed to estimate the HRs for the risk of death, HS, and IS in each MMD subgroup between the bypass (direct and indirect) and conservative groups (Table 1 and eTable 4 in Supplement 1). Compared with conservative management, bypass was associated with reduced risk of death in all MMD (adjusted HR [AHR], 0.68 [95% CI, 0.61-0.75]); a reduced risk of death (AHR, 0.50 [95% CI, 0.41-0.61]) and HS (AHR, 0.36 [95% CI, 0.30-0.40]) in hemorrhagic MMD; reduced risk of IS in ischemic MMD (AHR, 0.55 [95% CI, 0.37-0.81]); and reduced risk of death in asymptomatic MMD (AHR, 0.74 [95% CI, 0.66-0.84]). However, bypass was associated with an increased risk of HS in asymptomatic MMD (AHR, 1.76 [95% CI, 1.56-2.00]) and all MMD (AHR, 1.13 [95% CI, 1.05-1.22]). The results suggest that bypass surgery may be more effective than conservative management in reducing death in adult MMD and HS in the hemorrhagic MMD. However, the association of bypass with IS appears to be less clear, except in the ischemic MMD (AHR, 0.55 [95% CI, 0.37-0.81]), and the risk of HS may increase in asymptomatic MMD (AHR, 1.76 [95% CI, 1.56-2.00]).

**Table 1.** Outcomes After Bypass Surgery in Each MMD Subgroup

Subgroup by management strategy and covariates	Outcome					
	Death		Hemorrhagic stroke		Ischemic stroke	
	AHR (95% CI)	*P* value	AHR (95% CI)	*P* value	AHR (95% CI)	*P* value
**All MMD**						
Management strategy bypass vs conservative						
Bypass	0.68 (0.61-0.75)	<.001	1.13 (1.05-1.22)	.002	1.02 (0.86-1.21)	.84
Conservative	1 [Reference]	NA	1 [Reference]	NA	1 [Reference]	NA
Age	1.02 (1.02-1.03)	<.001	1.02 (1.02-1.03)	<.001	1.03 (1.02-1.03)	<.001
Male sex	1.42 (1.32-1.53)	<.001	0.92 (0.86-0.98)	.01	1.14 (0.99-1.31)	.06
Hypertension	0.85 (0.77-0.93)	<.001	1.06 (0.99-1.15)	.11	1.10 (0.94-1.29)	.26
Type 2 diabetes	1.44 (1.28-1.61)	<.001	1.00 (0.90-1.10)	.96	1.47 (1.23-1.77)	<.001
Dyslipidemia	0.65 (0.59-0.72)	<.001	0.54 (0.50-0.59)	<.001	1.08 (0.92-1.28)	.34
Management strategy type of bypass vs conservative						
Direct	0.64 (0.56-0.73)	<.001	1.03 (0.94-1.13)	.48	1.00 (0.81-1.23)	>.99
Indirect	0.73 (0.63-0.85)	<.001	1.31 (1.17-1.46)	<.001	1.05 (0.81-1.37)	.71
Conservative	1 [Reference]	NA	1 [Reference]	NA	1 [Reference]	NA
Age	1.02 (1.02-1.03)	<.001	1.02 (1.02-1.03)	<.001	1.03 (1.02-1.03)	<.001
Male sex	1.43 (1.32-1.54)	<.001	.92 (0.86-0.98)	.01	1.14 (0.99-1.31)	.06
Hypertension	0.85 (0.77-0.93)	<.001	1.06 (0.98-1.14)	.12	1.10 (0.94-1.29)	.26
Type 2 diabetes	1.44 (1.28-1.61)	<.001	1.00 (0.9-1.1)	.98	1.47 (1.23-1.77)	<.001
Dyslipidemia	0.65 (0.59-0.72)	<.001	0.54 (0.50-0.59)	<.001	1.08 (0.92-1.28)	.33
**Hemorrhagic MMD**						
Management strategy, bypass vs conservative						
Bypass	0.50 (0.41-0.61)	<.001	0.36 (0.3-0.4)	<.001	1.13 (0.76-1.69)	.54
Conservative	1 [Reference]	NA	1 [Reference]	NA	1 [Reference]	NA
Age	1.02 (1.02-1.03)	<.001	1.00 (1.00-1.00)	.92	1.02 (1.01-1.04)	.003
Male sex	1.10 (0.96-1.27)	.16	0.91 (0.84-0.99)	.03	1.37 (0.98-1.91)	.07
Hypertension	0.77 (0.66-0.91)	.002	1.08 (0.98-1.18)	.11	0.83 (0.56-1.24)	.36
Type 2 diabetes	1.34 (1.08-1.66)	.007	1.03 (0.90-1.18)	.65	1.67 (1.04-2.66)	.03
Dyslipidemia	0.82 (0.67-1.00)	.04	0.90 (0.81-1.00)	.06	1.61 (1.05-2.45)	.03
Management strategy, type of bypass vs conservative						
Direct	0.39 (0.28-0.52)	<.001	0.35 (0.31-0.39)	<.001	1.17 (0.71-1.95)	.54
Indirect	0.63 (0.48-0.82)	<.001	0.39 (0.34-0.45)	<.001	1.09 (0.63-1.89)	.76
Conservative	1 [Reference]	NA	1 [Reference]	NA	1 [Reference]	NA
Age	1.02 (1.02-1.03)	<.001	1.00 (1.00-1.00)	.92	1.02 (1.01-1.04)	.003
Male sex	1.11 (0.97-1.27)	.14	0.92 (0.84-0.99)	.03	1.36 (0.97-1.91)	.07
Hypertension	0.77 (0.66- 0.91)	.001	1.08 (0.98-1.18)	.12	0.83 (0.56-1.24)	.36
Type 2 diabetes	1.33 (1.07-1.65)	.01	1.03 (0.90-1.18)	.67	1.67 (1.04-2.67)	.03
Dyslipidemia	0.82 (0.68-1.00)	.052	0.90 (0.81-1.01)	.06	1.60 (1.05-2.45)	.03
**Ischemic MMD**						
Management strategy, bypass vs conservative						
Bypass	0.79 (0.40-1.54)	.48	0.76 (0.48-1.2)	.25	0.55 (0.37-0.81)	.002
Conservative	1 [Reference]	NA	1 [Reference]	NA	1 [Reference]	NA
Age	1.04 (1.02-1.06)	<.001	1.02 (1.00-1.03)	.04	1.01 (1.00-1.02)	.21
Male sex	1.75 (1.08-2.84)	.02	1.19 (0.83-1.70)	.34	1.18 (0.88-1.59)	.26
Hypertension	0.91 (0.53-1.54)	.72	0.72 (0.48-1.08)	.11	1.16 (0.83-1.61)	.39
Type 2 diabetes	0.95 (0.51-1.74)	.86	0.96 (0.58-1.59)	.87	1.65 (1.14-2.37)	.01
Dyslipidemia	1.02 (0.60-1.75)	.94	0.76 (0.50-1.15)	.20	0.90 (0.65-1.24)	.51
Management strategy, type of bypass vs conservative						
Direct	0.81 (0.38-1.74)	.60	0.85 (0.51-1.40)	.51	0.52 (0.33-0.83)	.01
Indirect	0.72 (0.22-2.39)	.59	0.58 (0.25-1.35)	.20	0.60 (0.31-1.16)	.13
Conservative	1 [Reference]	NA	1 [Reference]	NA	1 [Reference]	NA
Age	1.04 (1.02-1.06)	<.001	1.02 (1.00-1.03)	.04	1.01 (1.00-1.02)	.20
Male sex	1.74 (1.07-2.83)	.02	1.17 (0.82-1.68)	.39	1.19 (0.88-1.60)	.25
Hypertension	0.91 (0.54-1.54)	.72	0.72 (0.48-1.08)	.11	1.15 (0.83-1.61)	.39
Type 2 diabetes	0.94 (0.51-1.74)	.85	0.95 (0.57-1.58)	.86	1.65 (1.15-2.37)	.01
Dyslipidemia	1.02 (0.59-1.74)	.95	0.75 (0.49-1.14)	.17	0.90 (0.65-1.25)	.54
**Asymptomatic MMD**						
Management strategy, bypass vs conservative						
Bypass	0.74 (0.66- 0.84)	<.001	1.76 (1.56-2.00)	<.001	1.01 (0.81-1.26)	.94
Conservative	1 [Reference]	NA	1 [Reference]	NA	1 [Reference]	NA
Age	1.02 (1.01-1.02)	<.001	1.03 (1.03-1.03)	<.001	1.03 (1.02-1.04)	<.001
Male sex	1.59 (1.46-1.75)	<.001	0.87 (0.77-0.98)	.02	1.02 (0.85-1.22)	.80
Hypertension	0.87 (0.77-0.97)	.01	0.98 (0.86-1.12)	.80	1.08 (0.89-1.33)	.44
Type 2 diabetes	1.56 (1.36-1.79)	<.001	1.21 (1.02-1.43)	.03	1.34 (1.06-1.69)	.01
Dyslipidemia	0.68 (0.60-0.77)	<.001	0.64 (0.55-0.74)	<.001	1.07 (0.87-1.31)	.54
Management strategy, type of bypass vs conservative						
Direct	0.75 (0.65-0.87)	<.001	1.71 (1.47-1.98)	<.001	0.99 (0.75-1.29)	.92
Indirect	0.73 (0.60-0.88)	.001	1.87 (1.56-2.25)	<.001	1.05 (0.74-1.49)	.77
Conservative	1 [Reference]	NA	1 [Reference]	NA	1 [Reference]	NA
Age	1.02 (1.01-1.02)	<.001	1.03 (1.03-1.03)	<.001	1.03 (1.02-1.03)	<.001
Male sex	1.59 (1.45-1.74)	<.001	0.87 (0.08-0.98)	.02	1.02 (0.85-1.22)	.82
Hypertension	0.87 (0.77-0.97)	.01	0.98 (0.86-1.12)	.78	1.08 (0.88-1.33)	.44
Diabetes	1.56 (1.36-1.79)	<.001	1.21 (1.02-1.44)	.03	1.34 (1.06-1.69)	.01
Dyslipidemia	0.68 (0.60-0.77)	<.001	0.64 (0.56-0.75)	<.001	1.07 (0.87-1.32)	.53

Abbreviations: AHR, adjusted hazard ratio; MMD, moyamoya disease; NA, not applicable.

Among the direct bypass, indirect bypass, and conservative management groups (Table 1 and eTable 4 in Supplement 1), both direct and indirect bypass were associated with a reduced risk of death in all MMD (AHRs, 0.64 [95% CI, 0.56-0.73], *P* < .001 for direct bypass and 0.73 [95% CI, 0.63-0.85], *P* < .001 for indirect bypass); a reduced risk of death (AHRs, 0.39 [95% CI, 0.28-0.52], *P* < .001 for direct bypass and 0.63 [95% CI, 0.48-0.82], *P* < .001 for indirect bypass) and HS (AHRs, 0.35 [95% CI, 0.31-0.39], *P* < .001 for direct bypass, and 0.39 [95% CI, 0.34-0.45], *P* < .001 for indirect bypass) in hemorrhagic MMD; and a reduced risk for death in asymptomatic MMD (AHR, 0.75 [95% CI, 0.65-0.87], *P* < .001 for direct bypass and 0.73 [95% CI, 0.60-0.88], *P* = .001 for indirect bypass). In ischemic MMD, only direct bypass was associated with reduced risk of IS (AHR, 0.52 [95% CI, 0.33-0.83]; *P* = .01). However, both direct (AHR, 1.71 [95% CI, 1.47-1.98]; *P* < .001) and indirect bypass (AHR, 1.87 [95% CI, 1.56-2.25]; *P* < .001) were associated with an increased risk of HS in asymptomatic MMD. Bypass was associated with an increased risk of HS in all MMD groups, especially with indirect bypass (AHR, 1.31 [95% CI, 1.17-1.46]; *P* < .001). Among other confounders, diabetes was associated with an increased risk of death and IS in most MMD subgroups (1.44 [95% CI, 1,28-1.61], *P* < .001 and 1.47 [95% CI, 1.23-1.77], *P* < .001, respectively, in all MMD; 1.33 [95% CI, 1.07-1.65], *P* = .01 and 1.67 [95% CI, 1.04-2.67], *P* = .03, respectively, in hemorrhagic MMD; 1.65 [95% CI, 1.15-2.37], *P* = .01 for IS in ischemic MMD; and 1.56 [95% CI, 1.36-1.79], *P* < .001 and 1.34 [95% CI, 1.06-1.69], *P* = .01, respectively, in asymptomatic MMD). Based on these results, direct and indirect bypass seem to have similar outcomes in most MMD subgroups compared with conservative management. However, only direct bypass was associated with a reduced risk of IS in ischemic MMD (AHR, 0.52 [95% CI, 0.33-0.83]; *P* = .01). The adverse events observed between direct and indirect bypass were comparable (eTable 5 in Supplement 1). These results suggest that the onset types might influence the choice of bypass methods.

### Sensitivity Analysis, Propensity Score–Matching Analysis, and Stratified Analysis

Sensitivity analysis was performed to validate the definitions of MMD subgroups for the risks of death, HS, and IS. All patients with MMD were reclassified into 3 subgroups according to their records for 1, 3, and 12 months prior to the occurrence of the MMD code. All results aligned with those derived using our primary methods (eTable 6 in Supplement 1).

**Figure 3.**
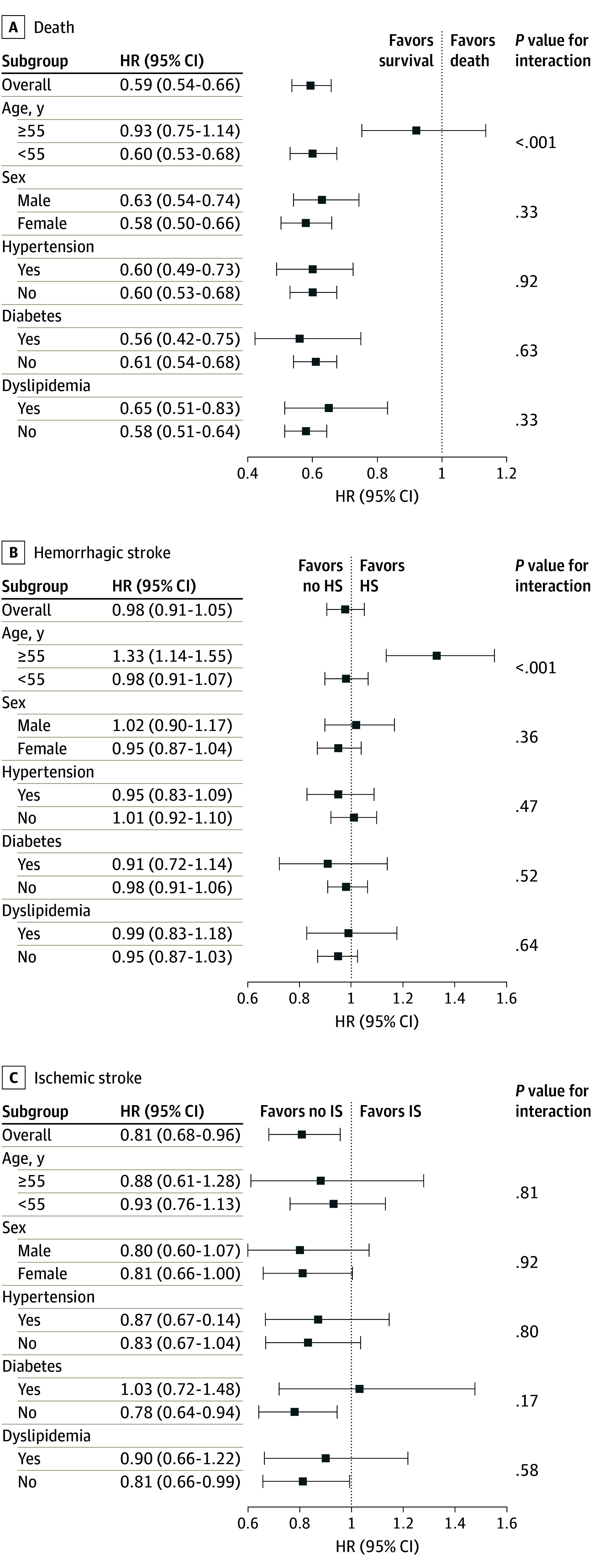
Hazard Ratios (HRs) for Patient Outcomes HS indicates hemorrhagic stroke; IS, ischemic stroke.

Regarding controlling covariates, the bypass group was propensity score matched 1:1 with the conservative group (Table 2). Baseline covariates were compared across the matched patient groups before and after matching. They were well balanced based on absolute mean differences with age, sex, hypertension, diabetes, and dyslipidemia (eFigure 3 in Supplement 1). The Kaplan-Meier curves were drawn to compare survival curves, and the log-rank test assessed the difference with a matched cohort. The association of a bypass with death, HS, and IS demonstrated similar trends observed in all cohorts as found in the primary analysis (eFigure 4 in Supplement 1). Furthermore, we stratified patients by each category of covariates. In most cases, age was a significant factor to interact with outcomes. Bypass was associated with a reduced risk of death compared with conservative management in patients younger than 55 years in all (AHR, 0.60 [95% CI, 0.53-0.68]; *P* < .001), ischemic (AHR, 0.34 [95% CI, 0.13-0.88]; *P* = .03), and asymptomatic (AHR, 0.69 [95% CI, 0.60-0.79]; *P* < .001) MMD, but an increased risk of HS in patients 55 years or older with ischemic MMD (AHR, 2.13 [95% CI, 1.10-4.16]; *P* = .03) (Figure 3 and eTable 7 in Supplement 1). When considering bypass surgery, patients should be carefully selected, particularly with regard to age.

**Table 2.** Outcomes in Each MMD Subgroup According to Management Modality With 1:1 Propensity Score–Matched Cohort

Management by MMD subgroup	No. of patients	Outcome						
		Death			Hemorrhagic stroke		Ischemic stroke	
		AHR (95% CI)	*P* value	AHR (95% CI)		*P* value	AHR (95% CI)	*P* value
All								
Conservative	4168	1 [Reference]	NA	1 [Reference]		NA	1 [Reference]	NA
Bypass	4186	0.70 (0.62- 0.79)	<.001	1.16 (1.05-1.28)		.003	1.06 (0.85-1.33)	.56
Hemorrhagic								
Conservative	669	1 [Reference]	NA	1 [Reference]		NA	1 [Reference]	NA
Bypass	669	0.45 (0.36- 0.57)	<.001	0.30 (0.27 -0.34)		<.001	1.56 (0.88-2.76)	.13
Ischemic								
Conservative	125	1 [Reference]	NA	1 [Reference]		NA	1 [Reference]	NA
Bypass	125	0.74 (0.34-1.62)	.45	0.79 (0.47- 1.35)		.39	0.55 (0.35- 0.86)	.009
Asymptomatic								
Conservative	3367	1 [Reference]	NA	1 [Reference]		NA	1 [Reference]	NA
Bypass	3367	0.77 (0.66- 0.89)	<.001	1.72 (1.45-2.03)		<.001	1.02 (0.77-1.35)	.92

Abbreviations: AHR, adjusted hazard ratio; MMD, moyamoya disease; NA, not applicable.

## Discussion

To our knowledge, this study is the largest population-based retrospective longitudinal cohort study of adult MMD demonstrating the outcomes of bypass surgery in terms of death, HS, and IS according to the onset types. We present the results of 19 700 patients with a median follow-up of 5.74 (IQR, 2.95-9.42) years. Bypass was associated with a reduced risk of death and HS in hemorrhagic MMD, reduced risk of IS in ischemic MMD, and reduced risk of death in asymptomatic MMD. However, bypass was associated with an increased risk of HS in asymptomatic MMD. Both direct and indirect bypass demonstrated similar effects, except that only direct bypass was associated with a reduced risk of IS in ischemic MMD. These findings emphasize the need for individualized management strategies tailored to different clinical presentations.

### Management of Asymptomatic MMD in Adults

Data on the long-term prognosis of conservative management for adult patients with MMD are limited. Some studies^17,18,19,20,21,22^ have described the outcomes of conservative treatment for patients with asymptomatic or hemodynamically stable MMD. In a series of 113 adult patients with MMD,^17^ the disease progression rate was reported to be approximately 20% over 6 years. The reported annual stroke rate in patients with conservatively managed adult MMD is 3.2% to 15.0%.^18,19,20,21^ In a recent multicenter study in Japan with 103 asymptomatic adult patients with MMD,^22^ the annual risk of stroke was 1.0% in the first 5 years, predominantly hemorrhage in nature. In the present study, the annual rates of HS and IS were 1.21 and 0.56 per 100 person-years, respectively, in patients with asymptomatic MMD and conservative management (eTable 8 in Supplement 1). Bypass surgery was associated with a reduced risk of death and IS, but an increased risk of HS in asymptomatic MMD. These findings may provide information on the long-term prognosis of adults with conservatively managed asymptomatic MMD and emphasize the need for careful consideration of individual patient characteristics and disease progression when determining the treatment strategy.

### Management of Ischemic and Hemorrhagic MMD in Adults

The treatment in patients with MMD consists of augmenting blood flow and relieving hemodynamic stress on moyamoya vessels to prevent future stroke.^23^ Bypass surgery is usually considered for patients with recurrent clinical symptoms due to apparent cerebral ischemia or decreased regional cerebral blood flow, vascular response, Suzuki stage,^24,25^ and reserve in perfusion studies^26^ and may help prevent further IS.^27^ In adults with ischemic MMD, a diminishment of moyamoya vessels has been observed on angiography after bypass surgery.^28^ It is likely that the dominant bypass flow reduces the burden on moyamoya vessels, which results in the relief of hemodynamic stress. Adults with ischemic MMD receiving medical management alone usually experience poor outcomes, with recurrent annual stroke rates of 20% to 50%.^29,30,31^ In reports examining adults with MMD treated with indirect bypass,^2,32,33,34,35,36^ 0 to 14.3% of patients experienced a postoperative stroke each year, and the weighted mean annual rate of stroke was 5.6%. In the present study, bypass surgery was associated with a reduced risk of death and IS, and IS risk remained after adjustment, which is consistent with previous reports.^27,37^

In adults with hemorrhagic MMD, the benefits of bypass remain unclear. Long-term hemodynamic stress to collateral vessels is thought to induce vascular abnormalities, such as microaneurysms, leading to hemorrhage.^1,38^ Diminishing of these abnormalities was observed after bypass surgery.^39,40^ Some authors suggest that the natural course and surgical effects depend on the initial hemorrhage site, and the patients with posterior hemorrhage face higher rebleeding risk and gain more benefit from surgery.^41^ Bypass surgery has been shown to reduce the subsequent HS rate by 12.5% to 20% (from an estimated occurrence rate of 30%-65%), although the evidence levels were not high.^13,14,42,43,44^ In contrast, some authors failed to observe the effectiveness of bypass to prevent further HS.^13,43^ Previous reports, however, were on retrospective studies that potentially contained various biases. The Japan Adult Moyamoya Disease trial was the first prospective, randomized clinical trial that aimed to compare the effect of surgical and conservative treatment for adult patients with hemorrhagic MMD.^11^ That trial showed that direct bypass surgery significantly decreased the rate of all adverse events and of rebleeding attacks, although the differences were only marginally significant. These results suggest that newly established bypass flow can influence the hemodynamic state of collateral vessels to prevent overstress. The present study may supplement the existing literature, with its larger sample size, longer follow-up period, and analysis of data from a national claims database. Bypass surgery was associated with a reduced risk of death and HS. These results support the idea that enhancing blood flow through bypass may positively influence the hemodynamic state of collateral vessels, potentially preventing future strokes.

### Direct and Indirect Bypass

There is considerable debate about the relative merits and shortcomings of direct and indirect bypass.^2^ Two meta-analyses^45,46^ demonstrated that indirect bypass was less effective in stroke reduction than direct bypass, with no significant difference in perioperative complications between the 2 modalities. Another meta-analysis on adult MMD^23^ demonstrated that combined and direct bypasses have significant benefits over indirect bypass for patients with late stroke and hemorrhage. Combined bypass was favored over indirect bypass due to more favorable clinical outcomes.^23^ A meta-analysis involving adults^47^ showed that direct bypass procedures are inferior in terms of quality-adjusted life-years at 4 years after surgery, suggesting that indirect and combination procedures may offer optimal results at long-term follow-up. In the present study, both direct and indirect methods were effective if surgery itself was effective on most occasions. Only direct bypass was associated with a reduced risk of IS in ischemic MMD. More detailed discussions are presented in the eDiscussion in Supplement 1. The choice between direct and indirect bypass involves a complex consideration of efficacy, technical challenges, and long-term outcomes, emphasizing the need for personalized management strategies.

### Strengths and Limitations

A strength of this study is the examination of nationwide, population-based data of MMD and the RID program, minimizing concerns about statistical power, selection bias (ie, compared with studies based on hospital records), and accessibility bias (all patients registered in the RID program become eligible for copayment reduction after their disease diagnosis). The large sample size, long follow-up periods, and unbiased measures (ie, surgery, incidence, and survival) ensure that the data are representative of patients with MMD.

This study also has some limitations. First, the retrospective design based on insurance claims data introduces information bias, lacking clinical or radiologic information such as hemodynamic and/or perfusion status,^27^ Suzuki grade, subtype (unilateral or bilateral involvement^48^), bleeding sites,^41^ laboratory test results, genetic information, and the date of death. These constraints may lead to underestimation or overestimation of results. Second, the reliance on administrative data may raise concerns regarding diagnostic reliability. However, the National Health Insurance Sharing Service has sent specific diagnostic criteria for RIDs to physicians for copayment reduction, and health care institutions are obligated to review physicians’ diagnoses before submitting the information. This process minimizes the likelihood of misclassification, ensuring the reliability of the diagnoses. Third, we cannot ascertain the exact registration rate of patients with MMD in the RID program and cannot completely exclude the possibility of some patients not registering. However, since every Korean health care institution is aware of the RID program and registering in the program offers patients copayment reduction, we assume the that high rate of registry for patients with MMD is accurate. Fourth, institutional bias, due to interinstitutional differences in diagnostic steps, surgical techniques, surgical success rates, the volume of center, and preferences, coupled with the absence of clear guidelines for bypass surgery, the result could be confounded by indication, limiting the possibility of drawing a definitive conclusion. Fifth, the observational design allowed patient allocation to bypass, influenced by physician judgment or patient preference. This nonrandom allocation introduces selection bias. Sixth, although addressed through propensity score matching, sensitivity analysis, and time-dependent survival analyses, unavailable or unmeasured confounding could persist, implying confounding bias. Seventh, surgical indication for hemorrhagic MMD may have changed substantially subsequent to the Japan Adult Moyamoya Trial.^11^ Our results showed an increased number of bypass procedures after 2014, introducing a potential temporal bias. Eighth, this study’s generalizability is confined to patients in South Korea, suggesting that cautious approach is necessary in extrapolating results to patients from non-Asian countries. Last, considering the peak age of adult MMD, our follow-up period may not be enough to identify all outcomes. Given these limitations, the results of our study should be interpreted with caution.

## Conclusions

The findings of this cohort study suggest that bypass surgery was associated with a reduced risk of death and HS, and both direct and indirect bypass had similar outcomes in most subgroups of adults with MMD. However, bypass surgery was associated with an increased risk of HS in asymptomatic MMD. These findings further suggest the importance of tailored treatment strategies for patients with adult MMD based on onset type and call for further research to optimize treatment approaches. Future investigations are needed to understand the natural course of MMD, as well as the hemodynamic and pathophysiological mechanisms influenced by bypass surgery. Large-scale, prospective, randomized clinical trials with extended follow-up periods are necessary to evaluate the long-term effects of bypass surgery for optimizing treatment strategies in patients with adult MMD.

## Notes

Supplement 1. **eMethods.** Participants, Comorbidity and Outcome Variables, and Statistical Analysis **eFigure 1.** Outcomes (Death, Hemorrhagic Stroke, and Ischemic Stroke) After Bypass Surgery in Each MMD Subgroup **eFigure 2.** Outcomes (Death, Hemorrhagic Stroke, and Ischemic Stroke) After Direct or Indirect Bypass Surgery in Each MMD Subgroup **eFigure 3.** Covariates Balance and Absolute Mean Difference Before and After Propensity Score Matching **eFigure 4.** Outcomes (Death, Hemorrhagic Stroke, and Ischemic Stroke) After Bypass Surgery in Each MMD Subgroup With 1:1 Propensity Score–Matched Cohort **eTable 1.** Baseline Characteristics of the Participants **eTable 2.** Baseline Characteristics of Participants in the Bypass vs Conservative Management Groups **eTable 3.** Trends in Bypass Surgical Procedures in Korea (2008-2020) **eTable 4.** Outcomes in Each MMD Subgroup According to Management Modality (Univariable Cox Proportional Hazards Regression Analysis) **eTable 5.** Adverse Events After Direct or Indirect Bypass Surgery **eTable 6.** Sensitivity Analysis Based on Operational Definition of MMD **eTable 7.** Bypass Outcomes Stratified by Age, Sex, Hypertension, Diabetes, and Dyslipidemia **eTable 8.** Incidence of Death, Hemorrhagic Stroke, and Ischemic Stroke in Each MMD Subgroup and According to Management Modality **eDiscussion.** General Stroke Risk Factors and Direct and Indirect Bypass
eReferences

Supplement 2.
Data Sharing Statement
