# Supplementary material for: Outcomes of Bypass Surgery in Adult Moyamoya Disease by Onset Type
Source: JAMA Netw Open. 2024 Jun 6;7(6):e2415102. doi: 10.1001/jamanetworkopen.2024.15102 (PMC11157360; doi:10.1001/jamanetworkopen.2024.15102)
Supplement: Supplement 1. — eMethods. Participants, Comorbidity and Outcome Variables, and Statistical Analysis eFigure 1. Outcomes (Death, Hemorrhagic Stroke, and Ischemic Stroke) After Bypass Surgery in Each MMD Subgroup eFigure 2. Outcomes (Death, Hemorrhagic Stroke, and Ischemic Stroke) After Direct or Indirect Bypass Surgery in Each MMD Subgroup eFigure 3. Covariates Balance and Absolute Mean Difference Before and After Propensity Score Matching eFigure 4. Outcomes (Death, Hemorrhagic Stroke, and Ischemic Stroke) After Bypass Surgery in Each MMD Subgroup With 1:1 Propensity Score–Matched Cohort eTable 1. Baseline Characteristics of the Participants eTable 2. Baseline Characteristics of Participants in the Bypass vs Conservative Management Groups eTable 3. Trends in Bypass Surgical Procedures in Korea (2008-2020) eTable 4. Outcomes in Each MMD Subgroup According to Management Modality (Univariable Cox Proportional Hazards Regression Analysis) eTable 5. Adverse Events After Direct or Indirect Bypass Surgery eTable 6. Sensitivity Analysis Based on Operational Definition of MMD eTable 7. Bypass Outcomes Stratified by Age, Sex, Hypertension, Diabetes, and Dyslipidemia eTable 8. Incidence of Death, Hemorrhagic Stroke, and Ischemic Stroke in Each MMD Subgroup and According to Management Modality eDiscussion. General Stroke Risk Factors and Direct and Indirect Bypass eReferences [file jamanetwopen-e2415102-s001.pdf]

Supplementary Online Content

Lim YC, Song J, Lee E. Outcomes of bypass surgery in adult moyamoya disease by onset type. *JAMA Netw Open*. 2024;7(6):e2415102.
doi:10.1001/jamanetworkopen.2024.15102

eMethods. Participants, Comorbidity and Outcome Variables, and Statistical Analysis

eFigure 1. Outcomes (Death, Hemorrhagic Stroke, and Ischemic Stroke) After Bypass Surgery in Each MMD Subgroup

eFigure 2. Outcomes (Death, Hemorrhagic Stroke, and Ischemic Stroke) After Direct or Indirect Bypass Surgery in Each MMD Subgroup

eFigure 3. Covariates Balance and Absolute Mean Difference Before and After Propensity Score Matching

eFigure 4. Outcomes (Death, Hemorrhagic Stroke, and Ischemic Stroke) After Bypass Surgery in Each MMD Subgroup With 1:1 Propensity Score–Matched Cohort

eTable 1. Baseline Characteristics of the Participants

eTable 2. Baseline Characteristics of Participants in the Bypass vs Conservative Management Groups

eTable 3. Trends in Bypass Surgical Procedures in Korea (2008-2020)

eTable 4. Outcomes in Each MMD Subgroup According to Management Modality (Univariable Cox Proportional Hazards Regression Analysis)

eTable 5. Adverse Events After Direct or Indirect Bypass Surgery

eTable 6. Sensitivity Analysis Based on Operational Definition of MMD

eTable 7. Bypass Outcomes Stratified by Age, Sex, Hypertension, Diabetes, and Dyslipidemia

eTable 8. Incidence of Death, Hemorrhagic Stroke, and Ischemic Stroke in Each MMD Subgroup and According to Management Modality

eDiscussion. General Stroke Risk Factors and Direct and Indirect Bypass

eReferences.

This supplementary material has been provided by the authors to give readers additional information about their work.

© 2024 Lim YC et al. *JAMA Network Open*.

eMethods. Participants, Comorbidity and Outcome Variables, and Statistical Analysis

Study participants

We improved the accuracy of patient identification by using the following operational definitions. The diagnosis of MMD was established if ICD code I67.5 and RID code V128 were newly recorded at least once in the database at hospital discharge or more than twice in the outpatient department. Both patients with bilateral and unilateral involvement were included in our data analysis.[1] The exclusion criteria were as follows: (1) age younger than 15 years[2]; (2) the history of direct or indirect bypass surgery before a diagnosis of MMD; (3) cardiac arrhythmia (I48) that may cause thromboembolic complications; (4) cancers (Cxx); (5) unstable angina or myocardial infarction (I20-I25) within the past 12 months; (6) bleeding diathesis (D65-69).

These groups based on onset-types had the following codes in their records during the six months prior to the occurrence of the MMD code: the hemorrhagic MMD group consisted of patients who had hemorrhagic stroke codes (I60, I61, or I62); the ischemic MMD group consisted of patients who had ischemic stroke codes (I63 or I64); and the asymptomatic MMD group consisted of patients who had none of the above diagnosis codes. An exception was made for cases involving both hemorrhage and ischemia, which were classified as the hemorrhagic type.

We categorized the patients according to management strategy based on treatment codes: direct bypass (S4661), indirect bypass (S4662), and conservative management (absence of both S4661 and S4662). Patients who underwent both direct and indirect bypass (combined) were classified to direct bypass group.

© 2024 Lim YC et al. *JAMA Network Open*.

Comorbidity and outcome variables

The primary outcome was the occurrence of death, and the secondary outcomes were the occurrence of HS (I60, I61 or I62) or IS (I63 or I64) based on ICD-10 codes. The death date was determined as the date of the last medical records when patients did not have any medical records for more than 6 months[3], and HS and IS dates were determined when the corresponding diagnosis codes occurred. We followed the included patients from a diagnosis date of MMD until the event date or the date of last medical records before the last observation date, December 31, 2021, whichever was earlier. Baseline comorbidities (demonstrated in Discussion 1 in Supplementary 1), were defined as conditions diagnosed within 1-year before the MMD diagnosis date using the following ICD-10 diagnostic codes: hypertension, I10-I15; diabetes mellitus (DM), E10-E14; dyslipidemia, E78[4-7]. To ensure an accurate diagnosis, we regarded the presence of comorbidities as any diagnosis with these codes occurring at least twice.[8] To evaluate the periprocedural complications between direct and indirect bypass, we defined adverse events as 1) hemorrhagic complication (T810, S064, S065); 2) wound infection (T813, T814); 3) seizure (G40) within one month after bypass surgery.[9,10]

Statistical Analysis

General characteristics are presented as the proportion, mean (SD) or median [IQR] after comparison with chi-square tests, analysis of variance (ANOVA) or Kruskal–Wallis tests among the hemorrhagic, ischemic, and asymptomatic MMD groups. Additionally, the characteristics were compared between the management within each MMD group. The incidence rates per 100 person-years for death, HS, and IS were obtained by dividing the number of patients with the specified outcomes by person-years at risk. The annual trend of bypass management and direct surgery in the overall bypass surgeries were examined and tested by the one-sided Cochran-Armitage Trend Test. Kaplan–Meier survival curves were

© 2024 Lim YC et al. *JAMA Network Open*.

generated to determine overall survivals from the date of MMD diagnosis to the date of outcomes and were compared using the log-rank test. The survival rates at 1- and 5-year were estimated. The proportional hazard assumption was tested by Schoenfeld residuals, and Cox proportional hazards regression models were adopted to calculate hazard ratios (HRs) and 95% confidence intervals (CIs) between the management types: bypass vs. conservative, direct vs. indirect vs. conservative. Crude (simple) and adjusted (age at diagnosis, sex, hypertension, DM, and dyslipidemia) models were used in these analyses.[4-7]

The sensitivity analysis was performed regarding the definition of subgroups based on their onset-types. All MMD patients were classified into three subgroups according to their records six months before the MMD code, and MMD subgroups were defined accordingly. However, the subgroups can be differently identified depending on the observation period before the MMD code. Thus, we variously defined subcohorts using records of 1 month, 3 months, and 12 months before the MMD code and compared its results to the primary analysis results. To control covariate effects, we additionally performed propensity-score matching analysis using the radius method to balance covariates across groups and reduce selection bias in the observational study. The propensity score was estimated using a logistic regression model with five matching variables: age, sex, hypertension, DM, and dyslipidemia. Based on these propensity scores, the bypass and conservative management groups were matched in a 1:1 ratio on an allowable absolute mean difference (0.2). The standardized mean differences were computed to measure the balance of covariates between groups before and after propensity-score matching. The Cox proportional hazard regression model was applied to compare the effect of management on each outcome with the matched cohort and to see the consistency of results. We also performed stratified analyses to examine the association of interests at the different levels of potential confounding factors. We explored the interaction between

© 2024 Lim YC et al. *JAMA Network Open*.

management groups and covariates and separated participants by the various categories of covariates: age (< 55 years, ≥ 55 years), sex (men, women), hypertension (yes, no), DM (yes, no), and dyslipidemia (yes, no). We investigated the significance of interaction terms and HRs and disclosed any modified management effects from primary analysis results.

Any missing data were excluded from the analysis. All statistical analyses were 2-sided, and $p<0.05$ was considered to indicate statistical significance. All analyses were performed using SAS Enterprise Guide version 6.3 (SAS Institute, Cary, NC). All plots were drawn with R version 4.1.2 (R Foundation for Statistical Computing, Vienna, Austria).

© 2024 Lim YC et al. *JAMA Network Open*.

eFigure 1. Outcomes (Death, Hemorrhagic Stroke, and Ischemic Stroke) After Bypass Surgery in Each MMD Subgroup

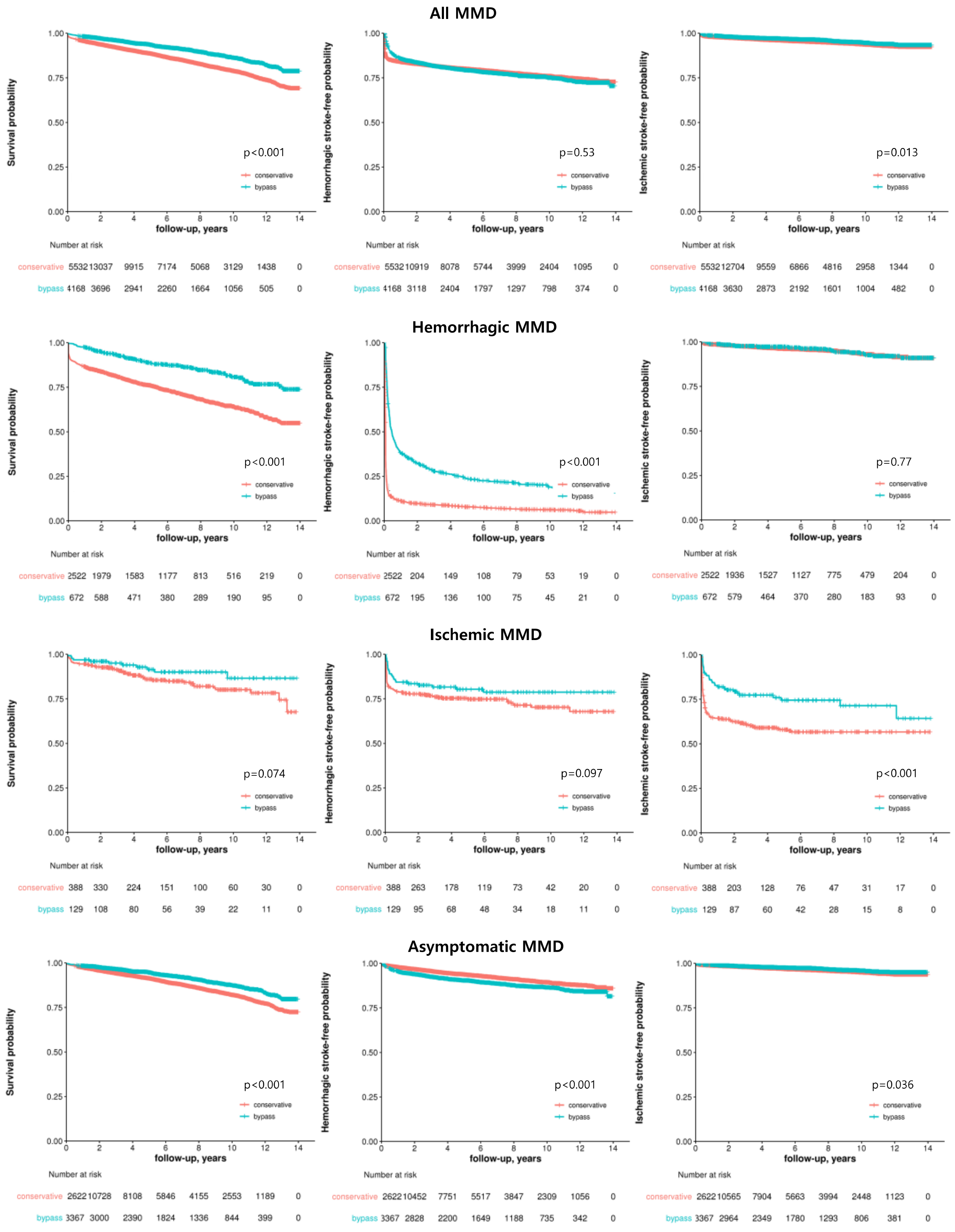

MMD, moyamoya disease; bypass, bypass surgery including both direct and indirect approaches

© 2024 Lim YC et al. *JAMA Network Open*.

eFigure 2. Outcomes (Death, Hemorrhagic Stroke, and Ischemic Stroke) After Direct or Indirect Bypass Surgery in Each MMD Subgroup

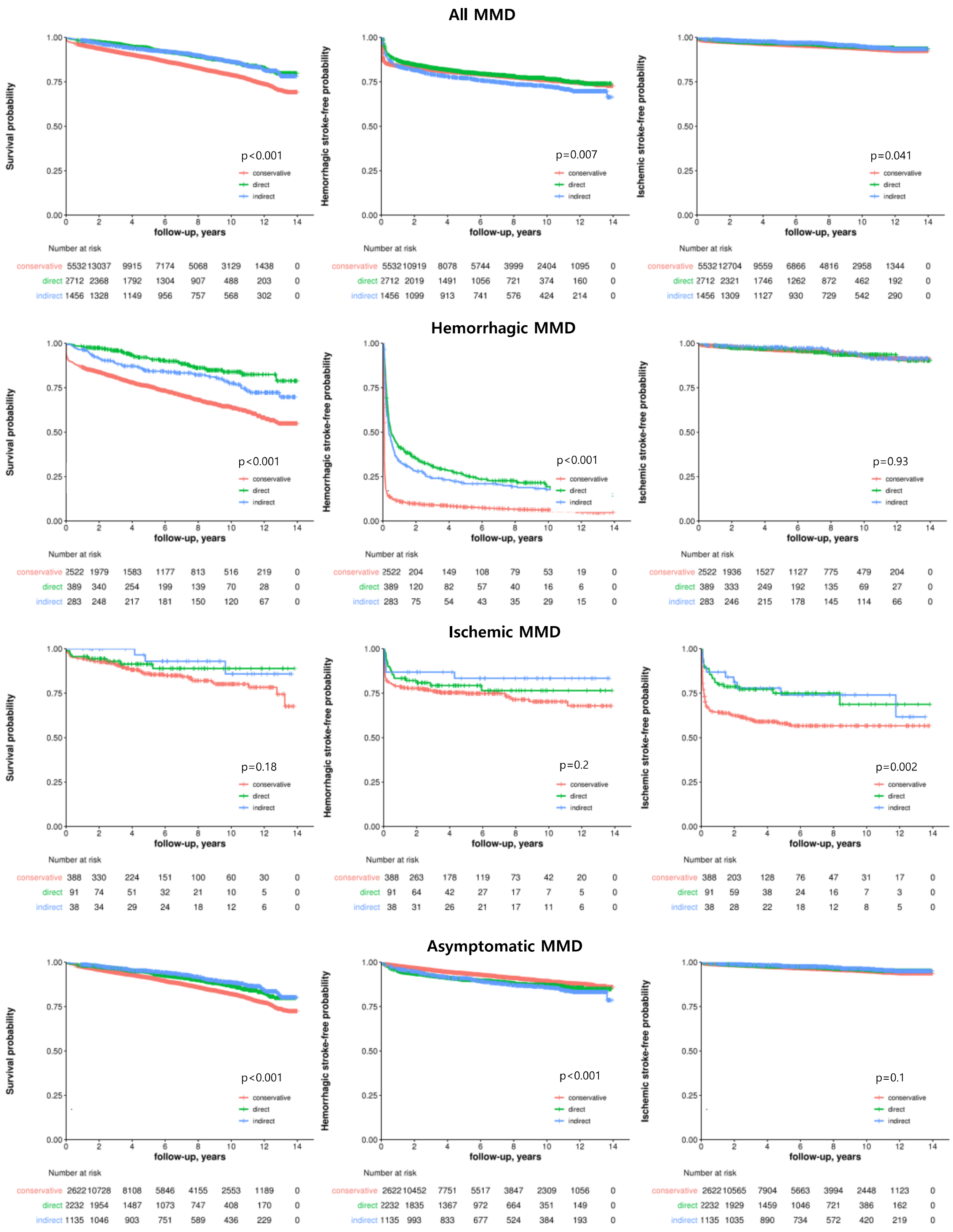

MMD, moyamoya disease; bypass, bypass surgery including both direct and indirect approaches.

© 2024 Lim YC et al. *JAMA Network Open*.

eFigure 3. Covariates Balance and Absolute Mean Difference Before and After Propensity Score Matching

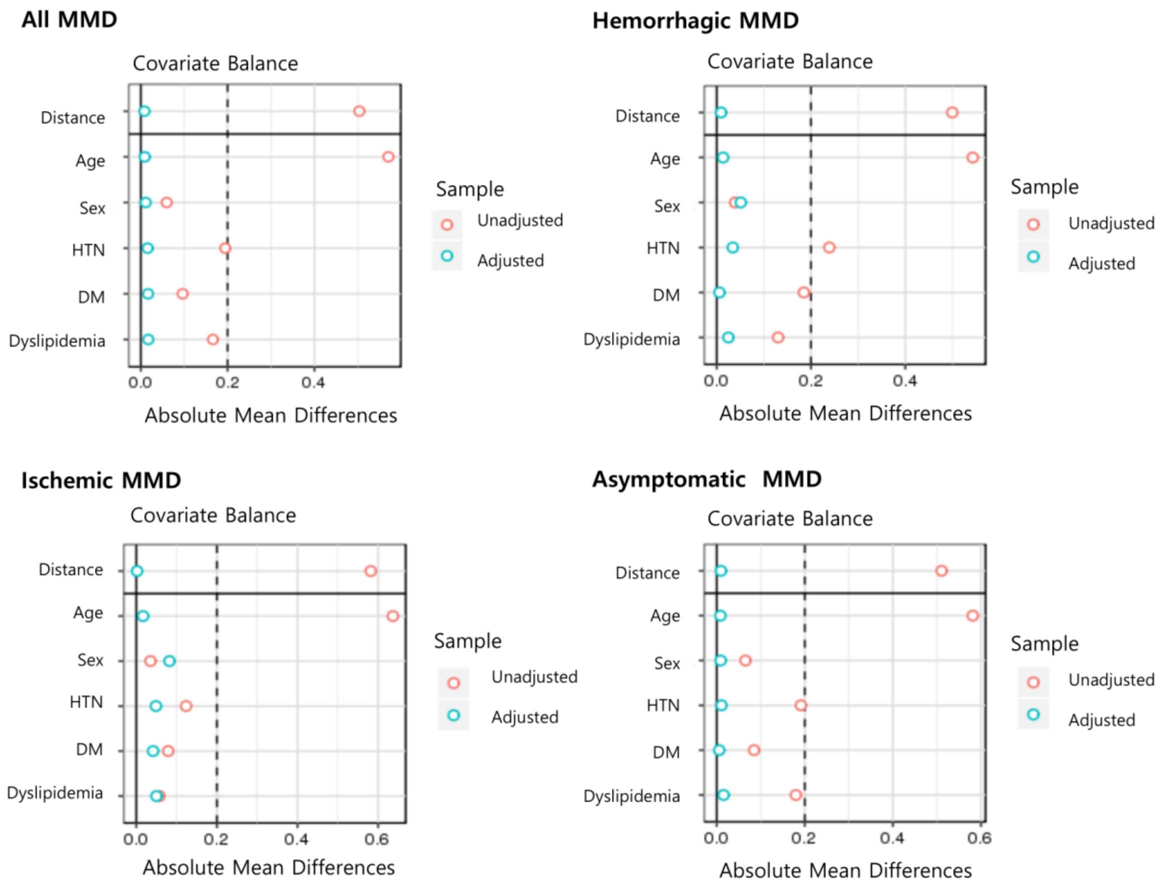

MMD, moyamoya disease; HTN, hypertension; DM, diabetes mellitus

© 2024 Lim YC et al. *JAMA Network Open*.

eFigure 4. Outcomes (Death, Hemorrhagic Stroke, and Ischemic Stroke) After Bypass Surgery in Each MMD Subgroup With 1:1 Propensity Score–Matched Cohort

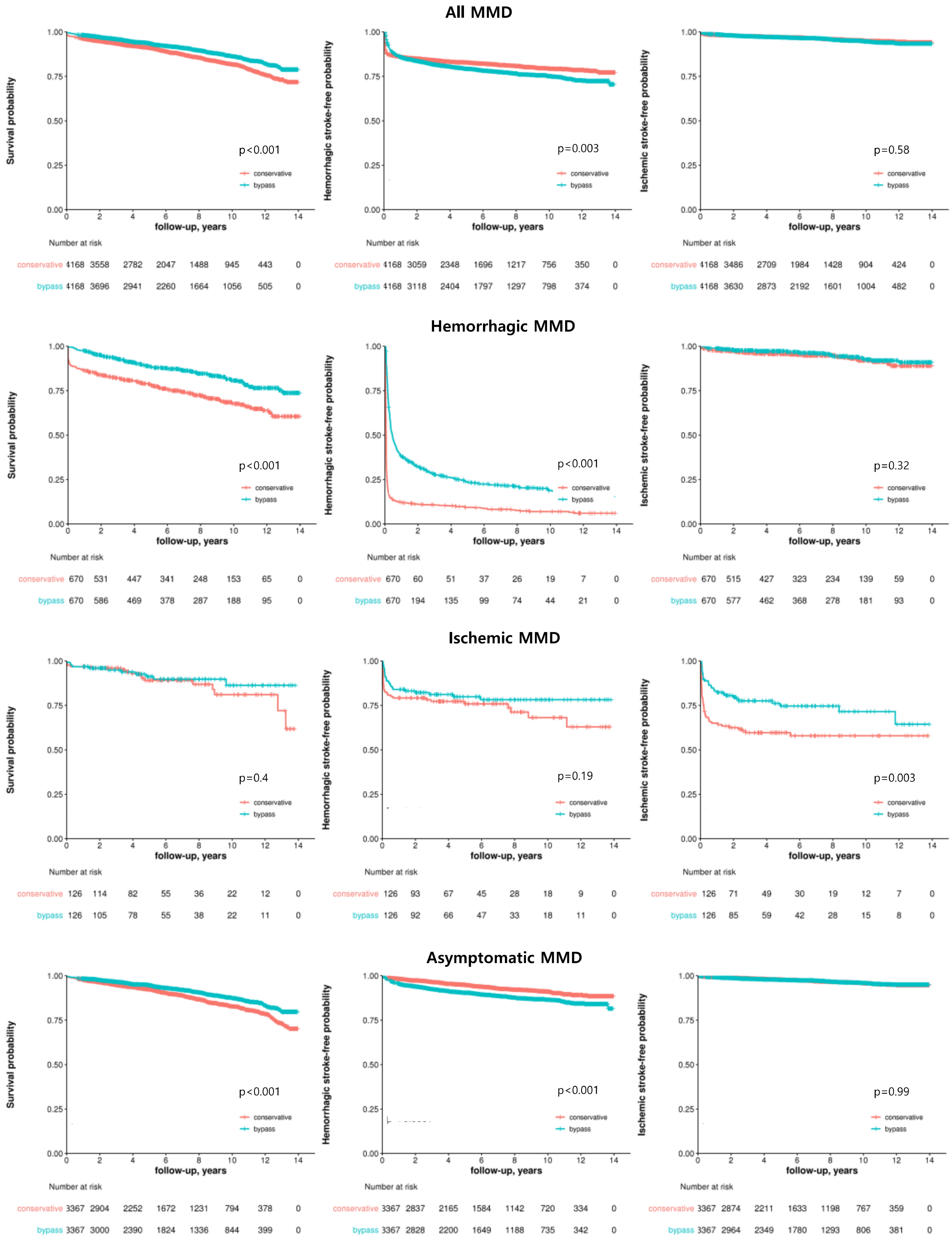

MMD, moyamoya disease; bypass, bypass surgery including both direct and indirect approaches.

© 2024 Lim YC et al. *JAMA Network Open*.

eTable 1. Baseline Characteristics of the Participants

		All MMD	Hemorrhagic MMD	Ischemic MMD	Asymptomatic MMD	p
**N (%)**		19700 (100)	3194 (16.21)	517 (2.62)	15989 (81.16)	
**Age, years, mean(std)**		45.43 (14.98)	48.24 (13.34)	49.71 (14)	44.73 (15.24)	<0.001
**Age group, N (%)**	15-19	1107 (5.62)	67 (2.1)	7 (1.35)	1033 (6.46)	<0.001
	20-24	935 (4.75)	88 (2.76)	13 (2.51)	834 (5.22)	
	25-29	1195 (6.07)	143 (4.48)	23 (4.45)	1029 (6.44)	
	30-34	1572 (7.98)	205 (6.42)	27 (5.22)	1340 (8.38)	
	35-39	2015 (10.23)	296 (9.27)	48 (9.28)	1671 (10.45)	
	40-44	2174 (11.04)	401 (12.55)	63 (12.19)	1710 (10.69)	
	45-49	2524 (12.81)	454 (14.21)	84 (16.25)	1986 (12.42)	
	50-54	2520 (12.79)	497 (15.56)	63 (12.19)	1960 (12.26)	
	55-59	2170 (11.02)	405 (12.68)	62 (11.99)	1703 (10.65)	
	60-64	1567 (7.95)	295 (9.24)	49 (9.48)	1223 (7.65)	
	65-69	946 (4.8)	179 (5.6)	34 (6.58)	733 (4.58)	
	70-74	541 (2.75)	102 (3.19)	23 (4.45)	416 (2.6)	
	75-79	289 (1.47)	40 (1.25)	14 (2.71)	235 (1.47)	
	80+	145 (0.74)	22 (0.69)	7 (1.35)	116 (0.73)	
**Sex, N (%)**						0.1
	Male	6934 (35.2)	1087 (34.03)	199 (38.49)	5648 (35.32)	
	Female	12766 (64.8)	2107 (65.97)	318 (61.51)	10341 (64.68)	
**Comorbidities, N (%)**						
Hypertension						<0.001
	No	13205 (67.03)	2103 (65.84)	285 (55.13)	10817 (67.65)	
	Yes	6495 (32.97)	1091 (34.16)	232 (44.87)	5172 (32.35)	
Diabetes mellitus						<0.001
	No	17046 (86.53)	2834 (88.73)	413 (79.88)	13799 (86.3)	
	Yes	2654 (13.47)	360 (11.27)	104 (20.12)	2190 (13.7)	
Dyslipidemia						<0.001
	No	14255 (72.36)	2581 (80.81)	326 (63.06)	11348 (70.97)	
	Yes	5445 (27.64)	613 (19.19)	191 (36.94)	4641 (29.03)	
**Management, N (%)**						0.101
	Conservative	15532 (78.84)	2522 (78.96)	388 (75.05)	12622 (78.94)	
	Bypass	4168 (21.16)	672 (21.04)	129 (24.95)	3367 (21.06)	
	Direct	2712 (13.77)	389 (12.18)	91 (17.6)	2232 (13.96)	
	Indirect	1456 (7.39)	283 (8.86)	38 (7.35)	1135 (7.1)	
**Outcomes, N (%)**						
Death						<0.001
	No	16857 (85.57)	2311 (72.35)	449 (86.85)	14097 (88.17)	
	Yes	2843 (14.43)	883 (27.65)	68 (13.15)	1892 (11.83)	
Hemorrhagic stroke						<0.001
	No	15590 (79.14)	485 (15.18)	393 (76.02)	14712 (92.01)	
	Yes	4110 (20.86)	2709 (84.82)	124 (23.98)	1277 (7.99)	
Ischemic stroke						<0.001
	No	18841 (95.64)	3052 (95.55)	330 (63.83)	15459 (96.69)	
	Yes	859 (4.36)	142 (4.45)	187 (36.17)	530 (3.31)	
**Continuous variables**						
Follow-up, yrs		5.74 [2.95, 9.42]	5.82 [2.72, 9.53]	4.76 [2.55, 8.41]	5.76 [3.00, 9.43]	<0.001
Diagnosis to surgery, days		56 [21, 161]	64 [19, 180]	44 [11, 123]	55 [21, 159]	0.104

Data are presented as N (%), mean (standard deviation (SD)), or median [interquartile range (IQR)]

MMD, moyamoya disease; yrs, years; Bypass, bypass surgery; conservative, conservative management.

© 2024 Lim YC et al. *JAMA Network Open*.

eTable 2. Baseline Characteristics of Participants in the Bypass vs Conservative Management Groups

		All MMD			Hemorrhagic MMD			Ischemic MMD			Asymptomatic MMD		
		Conservative	Bypass	p	Conservative	Bypass	p	Conservative	Bypass	p	Conservative	Bypass	p
**Age, years, mean(std)**		47.04 (14.98)	39.41 (13.38)	<0.001	49.73 (13.02)	42.65 (13.05)	<0.001	51.75 (13.79)	43.58 (12.83)	<0.001	46.36 (15.3)	38.61 (13.34)	<0.001
**Age group, N (%)**				<0.001			<0.001			0.001			<0.001
	15-19	745 (4.8)	362 (8.69)		34 (1.35)	33 (4.91)					709 (5.62)	324 (9.62)	
	20-24	613 (3.95)	322 (7.73)		47 (1.86)	41 (6.1)		8 (2.06)	5 (3.88)		558 (4.42)	276 (8.2)	
	25-29	813 (5.23)	382 (9.17)		94 (3.73)	49 (7.29)		14 (3.61)	9 (6.98)		705 (5.59)	324 (9.62)	
	30-34	1143 (7.36)	429 (10.29)		153 (6.07)	52 (7.74)		17 (4.38)	10 (7.75)		973 (7.71)	367 (10.9)	
	35-39	1466 (9.44)	549 (13.17)		205 (8.13)	91 (13.54)		32 (8.25)	16 (12.4)		1229 (9.74)	442 (13.13)	
	40-44	1635 (10.53)	539 (12.93)		313 (12.41)	88 (13.1)		42 (10.82)	21 (16.28)		1280 (10.14)	430 (12.77)	
	45-49	1953 (12.57)	571 (13.7)		351 (13.92)	103 (15.33)		62 (15.98)	22 (17.05)		1540 (12.2)	446 (13.25)	
	50-54	2067 (13.31)	453 (10.87)		414 (16.42)	83 (12.35)		45 (11.6)	18 (13.95)		1608 (12.74)	352 (10.45)	
	55-59	1873 (12.06)	297 (7.13)		332 (13.16)	73 (10.86)		50 (12.89)	12 (9.3)		1491 (11.81)	212 (6.3)	
	60-64	1404 (9.04)	163 (3.91)		259 (10.27)	36 (5.36)		46 (11.86)	3 (2.33)		1099 (8.71)	124 (3.68)	
	65-69	875 (5.63)	71 (1.7)		164 (6.5)	15 (2.23)		28 (7.22)	6 (4.65)		683 (5.41)	50 (1.49)	
	70-74	519 (3.34)	22 (0.53)		96 (3.81)	6 (0.89)		22 (5.67)	1 (0.78)		401 (3.18)	15 (0.45)	
	75-79	283 (1.82)	6 (0.14)		38 (1.51)	2 (0.3)		14 (3.61)	0 (0)		231 (1.83)	4 (0.12)	
	80+	143 (0.92)	2 (0.05)		22 (0.87)	0 (0)		6 (1.55)	1 (0.78)		115 (0.91)	1 (0.03)	
**Sex, N (%)**				0.0008			0.3742			0.7298			0.001
	Male	5559 (35.79)	1375 (32.99)		868 (34.42)	219 (32.59)		151 (38.92)	48 (37.21)		4540 (35.97)	1108 (32.91)	
	Female	9973 (64.21)	2793 (67.01)		1654 (65.58)	453 (67.41)		237 (61.08)	81 (62.79)		8082 (64.03)	2259 (67.09)	
**Continuous variables**													
Follow-up, yrs		5.55 (2.80, 9.20)	6.62 (3.50, 10.05)	<0.001	5.55 (2.50, 9.25)	7.05 (3.47, 10.51)	<0.001	4.67 (2.64, 8.35)	5.13 (2.32, 8.55)	0.5558	5.57 (2.86, 9.22)	6.58 (3.55, 10.01)	<0.001

© 2024 Lim YC et al. *JAMA Network Open*.

Diagnosis to surgery, days			56 (21, 161)			64 (19, 180)			44 (11, 123)			55 (21, 159)	

		All MMD			Hemorrhagic MMD			Ischemic MMD			Asymptomatic MMD		
		Conservative	Bypass	p	Conservative	Bypass	p	Conservative	Bypass	p	Conservative	Bypass	p
**Comorbidities, N (%)**													
Hypertension				<0.001			<0.001			0.2289			<0.001
	No	10130 (65.22)	3075 (73.78)		1605 (63.64)	498 (74.11)		208 (53.61)	77 (59.69)		8317 (65.89)	2500 (74.25)	
	Yes	5402 (34.78)	1093 (26.22)		917 (36.36)	174 (25.89)		180 (46.39)	52 (40.31)		4305 (34.11)	867 (25.75)	
Diabetes mellitus				<0.001			0.0004			0.4546			<0.001
	No	13340 (85.89)	3706 (88.92)		2212 (87.71)	622 (92.56)		307 (79.12)	106 (82.17)		10821 (85.73)	2978 (88.45)	
	Yes	2192 (14.11)	462 (11.08)		310 (12.29)	50 (7.44)		81 (20.88)	23 (17.83)		1801 (14.27)	389 (11.55)	
Dyslipidemia				<0.001			0.0059			0.5757			<0.001
	No	11012 (70.9)	3243 (77.81)		2013 (79.82)	568 (84.52)		242 (62.37)	84 (65.12)		8757 (69.38)	2591 (76.95)	
	Yes	4520 (29.1)	925 (22.19)		509 (20.18)	104 (15.48)		146 (37.63)	45 (34.88)		3865 (30.62)	776 (23.05)	
**Outcomes, N, (%)**													
Death				<0.001			<0.001			0.0728			<0.001
	No	13117 (84.45)	3740 (89.73)		1744 (69.15)	567 (84.38)		331 (85.31)	118 (91.47)		11042 (87.48)	3055 (90.73)	
	Yes	2415 (15.55)	428 (10.27)		778 (30.85)	105 (15.63)		57 (14.69)	11 (8.53)		1580 (12.52)	312 (9.27)	
Hemorrhagic stroke,				0.0622			<0.001			0.1574			<0.001
	No	12335 (79.42)	3255 (78.1)		334 (13.24)	151 (22.47)		289 (74.48)	104 (80.62)		11712 (92.79)	3000 (89.1)	
	Yes	3197 (20.58)	913 (21.9)		2188 (86.76)	521 (77.53)		99 (25.52)	25 (19.38)		910 (7.21)	367 (10.9)	
Ischemic stroke,				0.1296			0.5105			0.0019			0.172
	No	14837 (95.53)	4004 (96.07)		2413 (95.68)	639 (95.09)		233 (60.05)	97 (75.19)		12191 (96.59)	3268 (97.06)	
	Yes	695 (4.47)	164 (3.93)		109 (4.32)	33 (4.91)		155 (39.95)	32 (24.81)		431 (3.41)	99 (2.94)	
				0.001			0.001						0.001

© 2024 Lim YC et al. *JAMA Network Open*.

Data are presented as n (%), mean (standard deviation (SD)), or median [interquartile range (IQR)]; MMD, moyamoya disease; yrs, years; Bypass, bypass surgery; conservative, conservative management.

© 2024 Lim YC et al. *JAMA Network Open*.

eTable 3. Trends in Bypass Surgical Procedures in Korea (2008-2020)

		year													Total	p for trend
		2008	2009	2010	2011	2012	2013	2014	2015	2016	2017	2018	2019	2020		
All MMD	Conservative	949 (76.22)	961 (75.67)	954 (76.32)	1046 (76.91)	1041 (75.65)	1119 (78.25)	1074 (78.05)	1151 (79.71)	1364 (81.34)	1374 (79.33)	1373 (79.04)	1599 (82.59)	1527 (81.79)	15532	<0.001
	Bypass	296 (23.78)	309 (24.33)	296 (23.68)	314 (23.09)	335 (24.35)	311 (21.75)	302 (21.95)	293 (20.29)	313 (18.66)	358 (20.67)	364 (20.96)	337 (17.41)	340 (18.21)	4168	
	Direct	120 (40.54)	127 (41.1)	146 (49.32)	171 (54.46)	237 (70.75)	213 (68.49)	221 (73.18)	185 (63.14)	226 (72.2)	260 (72.63)	281 (77.2)	265 (78.64)	260 (76.47)	2712	<0.001
	Indirect	176 (59.46)	182 (58.9)	150 (50.68)	143 (45.54)	98 (29.25)	98 (31.51)	81 (26.82)	108 (36.86)	87 (27.8)	98 (27.37)	83 (22.8)	72 (21.36)	80 (23.53)	1456	
	Total	1245	1270	1250	1360	1376	1430	1376	1444	1677	1732	1737	1936	1867	19700	
Hemorrhagic MMD	Direct	24 (38.1)	9 (14.52)	22 (39.29)	29 (54.72)	41 (65.08)	34 (72.34)	39 (76.47)	23 (52.27)	19 (57.58)	33 (68.75)	49 (79.03)	35 (76.09)	32 (72.73)	389	<0.001
	Indirect	39 (61.9)	53 (85.48)	34 (60.71)	24 (45.28)	22 (34.92)	13 (27.66)	12 (23.53)	21 (47.73)	14 (42.42)	15 (31.25)	13 (20.97)	11 (23.91)	12 (27.27)	283	
	Total	63	62	56	53	63	47	51	44	33	48	62	46	44	672	

Numbers are presented as N (%); Bypass, bypass surgery; Conservative, conservative management.

© 2024 Lim YC et al. *JAMA Network Open*.

eTable 4. Outcomes in Each MMD Subgroup According to Management Modality (Univariable Cox Proportional Hazards Regression Analysis)

Management	Death		Hemorrhagic stroke		Ischemic stroke	
	HR (95% CI)	p	HR (95% CI)	p	HR (95% CI)	p
**All MMD**						
Bypass	0.59 (0.54-0.66)	<0.001	0.98 (0.91-1.05)	0.519	0.81 (0.68-0.96)	0.01
Conservative	Ref.		Ref.		Ref.	
Direct	0.58 (0.51-0.66)	<0.001	0.90 (0.82-0.99)	0.025	0.83 (0.67-1.02)	0.07
Indirect	0.62 (0.53-0.72)	<0.001	1.11 (1-1.24)	0.056	0.77 (0.59-1)	0.05
Conservative	Ref.		Ref.		Ref.	
**Hemorrhagic MMD**						
Bypass	0.44 (0.36-0.54)	<0.001	0.37 (0.33-0.4)	<0.001	0.94 (0.64-1.39)	0.77
Conservative	Ref.		Ref.		Ref.	
Direct	0.34 (0.25-0.46)	<0.001	0.34 (0.31-0.39)	<0.001	0.98 (0.6-1.62)	1
Indirect	0.56 (0.43-0.73)	<0.001	0.39 (0.34-0.45)	<0.001	0.90 (0.52-1.55)	0.71
Conservative	Ref.		Ref.		Ref.	
**Ischemic MMD**						
Bypass	0.56 (0.29-1.07)	0.078	0.69 (0.45-1.07)	0.098	0.51 (0.35-0.74)	<0.001
Conservative	Ref.		Ref.		Ref.	
Direct	0.63 (0.3-1.31)	0.216	0.76 (0.47-1.25)	0.28	0.50 (0.32-0.79)	0.003
Indirect	0.44 (0.14-1.39)	0.16	0.53 (0.23-1.22)	0.13	0.52 (0.274-0.98)	0.04
Conservative	Ref.		Ref.		Ref.	
**Asymptomatic MMD**						
Bypass	0.67 (0.59-0.75)	<0.001	1.44 (1.28-1.63)	<0.001	0.79 (0.64-0.99)	0.04
Conservative	Ref.		Ref.		Ref.	
Direct	0.70 (0.6-0.81)	<0.001	1.44 (1.24-1.66)	<0.001	0.82 (0.63-1.06)	0.13
Indirect	0.62 (0.51-0.75)	<0.001	1.45 (1.22-1.74)	<0.001	0.75 (0.54-1.06)	0.1
Conservative	Ref.		Ref.		Ref.	

MMD, moyamoya disease; bypass, bypass surgery; conservative, conservative management; Ref, reference; HR, hazard ratio; CI, confidence interval

© 2024 Lim YC et al. *JAMA Network Open*.

eTable 5. Adverse Events After Direct or Indirect Bypass Surgery

Adverse event	All				Hemorrhagic MMD				Ischemic MMD				Asymptomatic MMD			
	Bypass		Total	p	Bypass		Total	p	Bypass		Total	p	Bypass		Total	p
	Direct	Indirect			Direct	Indirect			Direct	Indirect			Direct	Indirect		
N (%)	2712(100)	1456(100)	4168(100)		389(100)	283(100)	672(100)		91(100)	38(100)	129		2232(100)	1135(100)	3367(100)	
Hemorrhagic complication, N (%)	33(1.22)	22(1.51)	55(1.32)	0.43	7(1.80)	11(3.89)	18(2.68)	0.1	2(2.20)	-	2(1.55)	1	24(1.08)	11(0.97)	35(1.04)	0.77
Wound infection, N (%)	82(3.02)	32(2.20)	114(2.74)	0.12	8(2.06)	7(2.47)	15(2.23)	0.72	-	-	-	-	74(3.32)	14(2.20)	99(2.94)	0.07
Seizure, N (%)	833(30.72)	480(32.97)	1313(31.50)	0.14	149(38.30)	128(45.23)	277(41.22)	0.07	30(32.97)	14(42.11)	46(35.66)	0.32	654(29.30)	336(29.60)	990(29.40)	0.86

MMD, moyamoya disease

© 2024 Lim YC et al. *JAMA Network Open*.

eTable 6. Sensitivity Analysis Based on Operational Definition of MMD

		1 month		3months		12 months	
		HR (95% CI)	p	HR (95% CI)	p	HR (95% CI)	p
**Death**							
**Hemorrhagic MMD**							
univariable	Bypass	0.42 (0.34-0.52)	<0.001	0.43 (0.35-0.53)	<0.001	0.44 (0.36-0.54)	<0.001
	Conservative	Ref.		Ref.		Ref.	
	Direct	0.32 (0.23-0.44)	<0.001	0.34 (0.25-0.46)	<0.001	0.34 (0.25-0.46)	<0.001
	Indirect	0.54 (0.41-0.7)	<0.001	0.55 (0.42-0.71)	<0.001	0.56 (0.44-0.73)	<0.001
	Conservative	Ref.		Ref.		Ref.	
multivariable	Bypass	0.48 (0.38-0.59)	<0.001	0.49 (0.4-0.61)	<0.001	0.50 (0.41-0.62)	<0.001
	Conservative	Ref.		Ref.		Ref.	
	Direct	0.36 (0.27-0.5)	<0.001	0.39 (0.29-0.53)	<0.001	0.39 (0.28-0.52)	<0.001
	Indirect	0.60 (0.46-0.79)	<0.001	0.61 (0.47-0.8)	<0.001	0.63 (0.49-0.82)	<0.001
	Conservative	Ref.		Ref.		Ref.	
**Ischemic MMD**							
univariable	Bypass	0.60 (0.3-1.18)	0.14	0.54 (0.27-1.053)	0.07	0.57 (0.31-1.05)	0.07
	Conservative	Ref.		Ref.		Ref.	
	Direct	0.66 (0.3-1.46)	0.3	0.58 (0.27-1.28)	0.18	0.58 (0.28-1.21)	0.15
	Indirect	0.49 (0.15-1.58)	0.23	0.46 (0.14-1.46)	0.18	0.55 (0.2-1.5)	0.24
	Conservative	Ref.		Ref.		Ref.	
multivariable	Bypass	0.80 (0.39-1.62)	0.53	0.76 (0.38-1.52)	0.43	0.81 (0.43-1.53)	0.51
	Conservative	Ref.		Ref.		Ref.	
	Direct	0.80 (0.35-1.8)	0.59	0.77 (0.34-1.72)	0.52	0.76 (0.36-1.61)	0.47
	Indirect	0.80 (0.24-2.66)	0.71	0.73 (0.22-2.392)	0.6	0.94 (0.33-2.66)	0.9
	Conservative	Ref.		Ref.		Ref.	
**Asymptomatic MMD**							
univariable	Bypass	0.67 (0.59-0.76)	<0.001	0.67 (0.59-0.75)	<0.001	0.67 (0.59-0.75)	<0.001
	Conservative	Ref.		Ref.		Ref.	
	Direct	0.70 (0.6-0.81)	<0.001	0.70 (0.6-0.81)	<0.001	0.70 (0.61-0.82)	<0.001
	Indirect	0.62 (0.52-0.75)	<0.001	0.63 (0.52-0.75)	<0.001	0.62 (0.51-0.74)	<0.001
	Conservative	Ref.		Ref.		Ref.	
multivariable	Bypass	0.75 (0.66-0.85)	<0.001	0.75 (0.66-0.84)	<0.001	0.74 (0.66-0.84)	<0.001
	Conservative	Ref.		Ref.		Ref.	
	Direct	0.76 (0.65-0.88)	<0.001	0.75 (0.65-0.87)	<0.001	0.76 (0.65-0.88)	<0.001
	Indirect	0.74 (0.61-0.89)	0.002	0.74 (0.61-0.89)	0.002	0.72 (0.6-0.88)	<0.001
	Conservative	Ref.		Ref.		Ref.	

© 2024 Lim YC et al. *JAMA Network Open*.

		1 month		3months		12 months	
		HR (95% CI)	p	HR (95% CI)	p	HR (95% CI)	p
**Hemorrhagic stroke**							
**Hemorrhagic MMD**							
univariable	Bypass	0.34 (0.31-0.37)	<0.001	0.35 (0.32-0.39)	<0.001	0.38 (0.34-0.42)	<0.001
	Conservative	Ref.		Ref.		Ref.	
	Direct	0.32 (0.28-0.36)	<0.001	0.34 (0.3-0.38)	<0.001	0.36 (0.32-0.41)	<0.001
	Indirect	0.37 (0.32-0.42)	<0.001	0.38 (0.33-0.44)	<0.001	0.41 (0.36-0.47)	<0.001
	Conservative	Ref.		Ref.		Ref.	
multivariable	Bypass	0.34 (0.3-0.37)	<0.001	0.36 (0.32-0.39)	<0.001	0.38 (0.34-0.42)	<0.001
	Conservative	Ref.		Ref.		Ref.	
	Direct	0.32 (0.28-0.36)	<0.001	0.34 (0.3-0.38)	<0.001	0.36 (0.32-0.41)	<0.001
	Indirect	0.36 (0.32-0.42)	<0.001	0.38 (0.33-0.44)	<0.001	0.41 (0.35-0.47)	<0.001
	Conservative	Ref.		Ref.		Ref.	
**Ischemic MMD**							
univariable	Bypass	0.59 (0.36-0.98)	0.04	0.62 (0.39-0.99)	0.05	0.69 (0.45-1.07)	0.1
	Conservative	Ref.		Ref.		Ref.	
	Direct	0.64 (0.36-1.12)	0.12	0.64 (0.37-1.11)	0.11	0.77 (0.47-1.25)	0.29
	Indirect	0.50 (0.2-1.23)	0.13	0.57 (0.25-1.3)	0.18	0.53 (0.23-1.21)	0.13
	Conservative	Ref.		Ref.		Ref.	
multivariable	Bypass	0.67 (0.4-1.11)	0.12	0.67 (0.41-1.09)	0.11	0.75 (0.48-1.19)	0.22
	Conservative	Ref.		Ref.		Ref.	
	Direct	0.71 (0.4-1.27)	0.25	0.70 (0.4-1.22)	0.21	0.84 (0.51-1.38)	0.49
	Indirect	0.56 (0.22-1.42)	0.22	0.60 (0.26-1.41)	0.24	0.57 (0.24-1.32)	0.19
	Conservative	Ref.		Ref.		Ref.	
**Asymptomatic MMD**							
univariable	Bypass	1.39 (1.23-1.56)	<0.001	1.43 (1.27-1.62)	<0.001	1.47 (1.3-1.66)	<0.001
	Conservative	Ref.		Ref.		Ref.	
	Direct	1.38 (1.19-1.59)	<0.001	1.42 (1.23-1.64)	<0.001	1.46 (1.27-1.69)	<0.001
	Indirect	1.41 (1.18-1.68)	0.001	1.46 (1.22-1.74)	<0.001	1.48 (1.24-1.77)	<0.001
	Conservative	Ref.		Ref.		Ref.	
multivariable	Bypass	1.70 (1.5-1.92)	<0.001	1.75 (1.54-1.98)	<0.001	1.80 (1.59-2.04)	<0.001
	Conservative	Ref.		Ref.		Ref.	
	Direct	1.64 (1.42-1.9)	<0.001	1.68 (1.45-1.95)	<0.001	1.74 (1.5-2.02)	<0.001
	Indirect	1.81 (1.51-2.16)	<0.001	1.87 (1.56-2.25)	<0.001	1.91 (1.59-2.3)	<0.001
	Conservative	Ref.		Ref.		Ref.	

© 2024 Lim YC et al. *JAMA Network Open*.

		1 month		3months		12 months	
		HR (95% CI)	p	HR (95% CI)	p	HR (95% CI)	p
**Ischemic stroke**							
**Hemorrhagic MMD**							
univariable	Bypass	0.94 (0.63-1.4)	0.77	0.92 (0.62-1.37)	0.68	0.97 (0.66-1.42)	0.86
	Conservative	Ref.		Ref.		Ref.	
	Direct	0.95 (0.57-1.58)	0.83	0.92 (0.55-1.54)	0.76	0.98 (0.59-1.61)	0.93
	Indirect	0.94 (0.55-1.62)	0.82	0.92 (0.53-1.58)	0.76	0.95 (0.56-1.61)	0.86
	Conservative	Ref.		Ref.		Ref.	
multivariable	Bypass	1.12 (0.75-1.69)	0.58	1.09 (0.72-1.63)	0.69	1.16 (0.78-1.73)	0.46
	Conservative	Ref.		Ref.		Ref.	
	Direct	1.12 (0.67-1.89)	0.66	1.08 (0.64-1.83)	0.76	1.17 (0.7-1.94)	0.55
	Indirect	1.12 (0.65-1.95)	0.68	1.09 (0.63-1.89)	0.77	1.16 (0.68-1.97)	0.6
	Conservative	Ref.		Ref.		Ref.	
**Ischemic MMD**							
univariable	Bypass	0.50 (0.33-0.74)	<0.001	0.52 (0.35-0.77)	0.001	0.53 (0.36-0.77)	0.001
	Conservative	Ref.		Ref.		Ref.	
	Direct	0.47 (0.29-0.76)	0.002	0.51 (0.32-0.81)	0.004	0.55 (0.36-0.85)	0.008
	Indirect	0.56 (0.29-1.06)	0.08	0.55 (0.29-1.03)	0.06	0.48 (0.24-0.93)	0.03
	Conservative	Ref.		Ref.		Ref.	
multivariable	Bypass	0.52 (0.34-0.78)	0.002	0.55 (0.37-0.82)	0.003	0.57 (0.39-0.84)	0.005
	Conservative	Ref.		Ref.		Ref.	
	Direct	0.47 (0.29-0.76)	0.002	0.52 (0.33-0.83)	0.006	0.58 (0.37-0.91)	0.02
	Indirect	0.65 (0.34-1.27)	0.21	0.63 (0.33-1.22)	0.17	0.54 (0.27-1.08)	0.08
	Conservative	Ref.		Ref.		Ref.	
**Asymptomatic MMD**							
univariable	Bypass	0.78 (0.63-0.97)	0.026	0.79 (0.64-0.99)	0.04	0.79 (0.64-0.99)	0.03
	Conservative	Ref.		Ref.		Ref.	
	Direct	0.83 (0.64-1.07)	0.147	0.83 (0.64-1.08)	0.16	0.81 (0.62-1.06)	0.12
	Indirect	0.72 (0.51-1.01)	0.054	0.74 (0.53-1.04)	0.08	0.76 (0.54-1.07)	0.11
	Conservative	Ref.		Ref.		Ref.	
multivariable	Bypass	1.01 (0.81-1.25)	0.952	1.02 (0.82-1.28)	0.85	1.01 (0.8-1.26)	0.95
	Conservative	Ref.		Ref.		Ref.	
	Direct	1.01 (0.78-1.31)	0.964	1.01 (0.78-1.31)	0.94	0.98 (0.75-1.28)	0.88
	Indirect	1.01 (0.71-1.43)	0.96	1.05 (0.74-1.48)	0.8	1.06 (0.75-1.51)	0.73

© 2024 Lim YC et al. *JAMA Network Open*.

Conservative	Ref.	Ref.	Ref.

MMD, moyamoya disease; Bypass, bypass surgery; Conservative, conservative management; Ref, reference; HR, hazard ratio; CI, confidence interval

© 2024 Lim YC et al. *JAMA Network Open*.

eTable 7. Bypass Outcomes Stratified by Age, Sex, Hypertension, Diabetes, and Dyslipidemia

	Death			Hemorrhagic stroke			Ischemic stroke		
	Interaction p	HR (95% CI)	p	Interaction p	HR (95% CI)	p	Interaction p	HR (95% CI)	p
**All MMD**									
Age ≥ 55	<0.001	0.92 (0.75-1.134)	0.4519	<0.001	1.33 (1.14-1.55)	<0.001	0.81	0.88 (0.61-1.28)	0.51
Age < 55		0.60 (0.53-0.68)	<0.001		0.98 (0.9-1.07)	0.62		0.93 (0.76-1.13)	0.46
Male	0.33	0.63 (0.54-0.74)	<0.001	0.36	1.02 (0.9-1.17)	0.718	0.92	0.80 (0.6-1.07)	0.13
Female		0.58 (0.5-0.66)	<0.001		0.95 (0.87-1.04)	0.26		0.81 (0.66-1.)	0.05
Hypertension	0.92	0.60 (0.49-0.73)	<0.001	0.47	0.95 (0.83-1.09)	0.458	0.8	0.87 (0.67-1.14)	0.33
No hypertension		0.60 (0.53-0.68)	<0.001		1.01 (0.92-1.1)	0.88		0.83 (0.67-1.04)	0.1
Diabetes mellitus	0.63	0.56 (0.42-0.75)	<0.001	0.52	0.91 (0.72-1.14)	0.4	0.17	1.03 (0.72-1.48)	0.87
No Diabetes mellitus		0.61 (0.54-0.68)	<0.001		0.98 (0.91-1.06)	0.67		0.78 (0.64-0.94)	0.01
Dyslipidemia	0.33	0.65 (0.51-0.83)	<0.001	0.64	0.99 (0.83-1.18)	0.89	0.58	0.90 (0.66-1.22)	0.49
No Dyslipidemia		0.58 (0.51-0.64)	<0.001		0.95 (0.87-1.03)	0.17		0.81 (0.66-0.99)	0.04
**Hemorrhagic MMD**									
Age ≥ 55	0.07	0.63 (0.44-0.91)	0.0137	0.1	0.44 (0.36-0.53)	<0.001	0.29	1.42 (0.72-2.83)	0.31
Age < 55		0.42 (0.33-0.54)	<0.001		0.34 (0.31-0.39)	<0.001		0.89 (0.55-1.43)	0.63
Male	0.28	0.51 (0.36-0.7)	<0.001	0.15	0.41 (0.35-0.49)	<0.001	0.22	1.25 (0.7-2.22)	0.45
Female		0.41 (0.31-0.53)	<0.001		0.34 (0.3-0.38)	<0.001		0.77 (0.45-1.32)	0.34
Hypertension	0.93	0.44 (0.29-0.66)	<0.001	0.26	0.41 (0.34-0.5)	<0.001	0.06	1.58 (0.87-2.84)	0.13
No hypertension		0.44 (0.35-0.55)	<0.001		0.35 (0.31-0.39)	<0.001		0.73 (0.43-1.22)	0.23
Diabetes mellitus	0.66	0.37 (0.19-0.73)	0.004	0.87	0.41 (0.29-0.57)	<0.001	0.7	1.10 (0.44-2.71)	0.84
No Diabetes mellitus		0.45 (0.36-0.56)	<0.001		0.36 (0.33-0.4)	<0.001		0.95 (0.61-1.46)	0.8
Dyslipidemia	0.23	0.58 (0.35-0.96)	0.04	0.16	0.44 (0.35-0.56)	<0.001	0.24	1.44 (0.71-2.95)	0.31

© 2024 Lim YC et al. *JAMA Network Open*.

No Dyslipidemia		0.42 (0.33-0.52)	<0.001		0.35 (0.31-0.39)	<0.001		0.86 (0.54-1.37)	0.52

	**Death**			**Hemorrhagic stroke**			**Ischemic stroke**		
	Interaction p	HR (95% CI)	p	Interaction p	HR (95% CI)	p	Interaction p	HR (95% CI)	p
**Ischemic MMD**									
Age ≥ 55	0.02	1.68 (0.69-4.08)	0.25	<0.001	2.13 (1.1-4.155)	0.0259	0.42	0.70 (0.34-1.45)	0.34
Age < 55		0.34 (0.13-0.88)	0.03		0.45 (0.25-0.804)	0.0071		0.49 (0.31-0.78)	0.002
Male	0.29	0.80 (0.35-1.84)	0.6	0.723	0.76 (0.39-1.483)	0.4244	0.68	0.46 (0.25-0.85)	0.01
Female		0.38 (0.14-1.09)	0.07		0.65 (0.36-1.164)	0.1471		0.55 (0.34-0.89)	0.01
Hypertension	0.31	0.75 (0.33-1.70)	0.48	0.27	0.93 (0.47-1.808)	0.8217	0.02	0.30 (0.16-0.58)	<0.001
No hypertension		0.37 (0.13-1.06)	0.06		0.55 (0.31-0.99)	0.0461		0.76 (0.47-1.23)	0.27
Diabetes mellitus	0.934	0.52 (0.12-2.3)	0.39	0.8	0.76 (0.26-2.24)	0.6164	0.70	0.59 (0.29-1.2)	0.15
No Diabetes mellitus		0.56 (0.28-1.16)	0.12		0.67 (0.41-1.081)	0.1006		0.49 (0.31-0.77)	0.002
Dyslipidemia	0.3	0.29 (0.07-1.25)	0.1	0.25	1.01 (0.47-2.131)	0.9884	0.51	0.42 (0.22-0.83)	0.01
No Dyslipidemia		0.71 (0.34-1.47)	0.35		0.58 (0.34-0.992)	0.0465		0.56 (0.35-0.9)	0.02
**Asymptomatic MMD**									
Age ≥ 55	0.02	1.00 (0.77-1.29)	0.97	0.68	1.69 (1.3-2.22)	0.001	0.19	0.65 (0.37-1.14)	0.13
Age < 55		0.69 (0.6-0.79)	<0.001		1.59 (1.38-1.83)	<0.001		0.98 (0.77-1.26)	0.88
Male	0.95	0.67 (0.56-0.81)	<0.001	0.73	1.49 (1.19-1.86)	0.0004	0.42	0.69 (0.46-1.03)	0.07
Female		0.68 (0.58-0.8)	<0.001		1.41 (1.22-1.63)	<0.001		0.84 (0.65-1.09)	0.19
Hypertension	0.82	0.66 (0.52-0.84)	<0.001	0.40	1.36 (1.09-1.69)	0.0064	0.7	0.88 (0.62-1.24)	0.45
No hypertension		0.67 (0.58-0.78)	<0.001		1.53 (1.32-1.77)	<0.001		0.80 (0.61-1.07)	0.13
Diabetes mellitus	0.75	0.65 (0.47-0.9)	0.009	0.76	1.38 (1-1.92)	0.0533	0.21	1.05 (0.66-1.67)	0.85
No Diabetes mellitus		0.68 (0.59-0.77)	<0.001		1.47 (1.29-1.67)	<0.001		0.76 (0.59-0.97)	0.03
Dyslipidemia	0.63	0.71 (0.54-0.94)	0.02	0.13	1.18 (0.89-1.56)	0.2512	0.79	0.85 (0.58-1.26)	0.43

© 2024 Lim YC et al. *JAMA Network Open*.

No Dyslipidemia	0.65 (0.57-0.75)	<0.001	1.51 (1.32-1.73)	<0.001	0.80 (0.62-1.05)	0.11

MMD, moyamoya disease; bypass, bypass surgery; conservative, conservative management; HR, hazard ratio; CI, confidence interval

© 2024 Lim YC et al. *JAMA Network Open*.

eTable 8. Incidence of Death, Hemorrhagic Stroke, and Ischemic Stroke in Each MMD Subgroup and According to Management Modality

	Death		Hemorrhagic Stroke		Ischemic stroke	
	IR (95% CI)	p	IR (95% CI)	p	IR (95% CI)	p
**All MMD**	2.28 (2.2,2.36)		4.00 (3.88,4.13)		0.71 (0.67,0.76)	
Conservative	2.51 (2.5, 2.52)	<0.001	4.05 (4.03, 4.06)	<0.001	0.75 (0.74, 0.76)	<0.001
Bypass	1.49 (1.5, 1.5)	<0.001	3.86 (3.83, 3.88)	<0.001	0.58 (0.58, 0.59)	<0.001
Direct	1.44 (1.4, 1.46)	<0.001	3.76 (3.73, 3.79)	<0.001	0.62 (0.61, 0.63)	<0.001
Indirect	1.56 (1.5, 1.58)	<0.001	4.00 (3.96, 4.05)	<0.001	0.53 (0.52, 0.55)	<0.001
**Hemorrhagic MMD**	4.44 (4.16,4.75)		77.51 (74.64,80.48)		0.74 (0.63,0.87)	
Conservative	5.15 (5.12, 5.19)	<0.001	115.05 (114.57, 115.53)	<0.001	0.75 (0.73, 0.76)	<0.001
Bypass	2.2 (2.16, 2.24)	<0.001	32.7 (32.42, 32.98)	<0.001	0.7 (0.68, 0.73)	<0.001
Direct	1.71 (1.7, 1.76)	<0.001	31.99 (31.63, 32.36)	<0.001	0.73 (0.7, 0.77)	<0.001
Indirect	2.74 (2.67, 2.81)	<0.001	33.66 (33.22, 34.1)	<0.001	0.67 (0.64, 0.71)	<0.001
**Ischemic MMD**	2.3 (1.81,2.92)		5.19 (4.35,6.19)		9.93 (8.6,11.46)	
Conservative	2.6 (2.53, 2.67)	<0.001	5.74 (5.62, 5.85)	<0.001	11.99 (11.8, 12.18)	<0.001
Bypass	1.44 (1.36, 1.53)	<0.001	3.77 (3.63, 3.92)	<0.001	5.42 (5.24, 5.61)	<0.001
Direct	1.67 (1.56, 1.79)	<0.001	4.67 (4.47, 4.89)	<0.001	5.93 (5.69, 6.19)	<0.001
Indirect	1.06 (0.94, 1.18)	0.35	2.34 (2.16, 2.54)	<0.001	4.56 (4.29, 4.85)	<0.001
**Asymptomatic MMD**	1.86 (1.77,1.94)		1.32 (1.25,1.39)		0.53 (0.49,0.58)	
Conservative	2.01 (2, 2.02)	<0.001	1.21 (1.2, 1.22)	<0.001	0.56 (0.56, 0.57)	<0.001
Bypass	1.34 (1.33, 1.36)	<0.001	1.71 (1.7, 1.73)	<0.001	0.44 (0.43, 0.44)	<0.001
Direct	1.38 (1.37, 1.4)	<0.001	1.74 (1.72, 1.77)	<0.001	0.46 (0.45, 0.47)	<0.001

© 2024 Lim YC et al. *JAMA Network Open*.

Indirect	1.28 (1.26, 1.31)	<0.001	1.67 (1.64, 1.69)	<0.001	0.4 (0.39, 0.42)	<0.001

MMD, moyamoya disease; Bypass, bypass surgery; Conservative, conservative management; IR, incidence rate; CI, confidence interval

© 2024 Lim YC et al. *JAMA Network Open*.

eDiscussion. General Stroke Risk Factors and Direct and Indirect Bypass

General stroke risk factors on adult MMD

The effect of general stroke risk factors on adult MMD has been relatively unexplored. While some studies suggest no association between hypertension and an increased risk of bleeding or rebleeding in MMD,[4,9] others demonstrated an increased risk of mortality.[6] Some authors suggest that hypertension and dyslipidemia may increase the risk of stroke in asymptomatic MMD patients. Another study identified DM as a predictor for stroke recurrence and its association with unfavorable outcomes after bypass surgery in ischemic MMD patients.[7] In the present study, DM was associated with an increased risk of IS across MMD subgroups and an increased risk of death in all, hemorrhagic, and asymptomatic MMD groups. Participants aged<55 group showed a greater reduced risk of death than the older group in most MMD subgroups. Patients should be carefully selected when considering bypass surgery, particularly regarding age.

Direct bypass and indirect bypass

There is considerable debate about the relative merits and shortcomings of direct and indirect bypass.[11] Historically, direct bypass has been used in adults for whom an immediate increase in blood flow to ameliorate the impaired cerebral perfusion represents a major benefit.[10] Following successful anastomosis between donor and recipient arteries, improvement in flow is achieved immediately after direct bypass.[12] Previous studies of direct bypass reported an annual stroke rate of .0-1.6%, with a weighted average annual stroke rate of 1.4%, which is considerably lower than the results seen with conservative treatment.[12-16] The relative risk reduction for IS by direct bypass is reportedly 7 .7%.[13] A study with 138 ischemic MMD patients showed that the subsequent stroke rate was comparable between the indirect and direct

© 2024 Lim YC et al. *JAMA Network Open*.

bypass groups, with direct bypass providing superior angiographic revascularization and indirect bypass providing superior improvement in hemodynamic reserve.[17] The clinical improvement tended to be greater in the direct bypass group, although the difference between the two bypass groups was not statistically significant. In the advanced stage of MMD, most of the cortical arteries tend to shrink to a small caliber and have more fragile vessel walls, making bypass procedures increasingly challenging. These characteristics of MMD vessels account for the technical difficulty of bypass, and requires a long learning curve to achieve proficiency. Additionally, postoperative hyperperfusion syndrome worsens the prognosis.[10]

The techniques required for indirect bypass are relatively easy, involving the placement of vascularized tissue supplied by the external carotid artery in direct contact with the brain. Even though it takes several weeks to augment cerebral blood flow and this approach is sometimes insufficient to establish sufficient collateral flow in adult patients, its technical ease leads to reduced operative time and invasiveness.[2,11,18] It may contribute to the well-documented decrease in adverse effects. A report of 220 patients found that direct procedures were more effective at preventing recurrent ischemic stroke than indirect procedures, although there was no difference in functional outcome.[19] In a series of 84 hemispheres, the authors demonstrated that direct and indirect techniques were equally effective at preventing future stroke, although direct procedures were superior in terms of achieving symptom improvement.[20] The authors of a single-center study report recommended indirect bypass, suggesting that this approach is sufficient for adults with MMD, after taking into consideration the complication rate, operating time, length of hospital stay, long-term revascularization, and clinical outcome.[19] In a comprehensive review of long-term follow-up data, indirect procedures were found to be significantly superior to direct procedures in providing quality-adjusted life years.[21] The divergent results from different studies probably reflect the

© 2024 Lim YC et al. *JAMA Network Open*.

heterogeneity in patient characteristics and patient selection among studies, as well as the varied lengths of follow-up.[18] Two meta-analyses demonstrated that indirect bypass was less effective in stroke reduction than direct bypass, with no significant difference in perioperative complications between the two modalities.[22,23] Another meta-analysis of 43 articles on adult MMD demonstrated that combined and direct bypasses have significant benefits over indirect bypass for patients suffering from late stroke and hemorrhage. Combined bypass was favored over indirect bypass due to more favorable clinical outcomes.[9] A meta-analysis of 33 studies involving adults showed that direct bypass procedures are inferior in terms of quality-adjusted life years at 4 years postoperatively, suggesting that indirect and combination procedures may offer optimal results at long-term follow-up.[21] In the present study, both direct and indirect methods were effective if surgery itself was effective on most occasions. Only direct bypass was associated with a reduced risk of IS in ischemic MMD. The adverse events observed between direct and indirect bypass were comparable that are comparable with previous meta-analysis .[22]Although direct bypass seems to be more effective in reducing the incidence of future stroke, the added risk of the procedure's technical difficulty, hyperperfusion syndrome, and subsequent higher morbidity rates than for indirect procedures and the increased rate of stenosis appear to outweigh the stroke benefit.[24] It is also possible that the risk of perioperative stroke from the delayed formation of collaterals following indirect bypass may be overstated. The reduced operative time and invasiveness, as well as the relative ease of indirect bypass, may contribute to the decrease in adverse effects with this procedure.[11]

© 2024 Lim YC et al. *JAMA Network Open*.

eReferences

1. Ahn HS, Kazmi SZ, Kang T, et al. Familial Risk for Moyamoya Disease Among First-Degree Relatives, Based on a Population-Based Aggregation Study in Korea. *Stroke*. Sep 2020;51(9):2752-2760. doi:10.1161/STROKEAHA.120.029251

2. Miyamoto S, Yoshimoto T, Hashimoto N, et al. Effects of extracranial-intracranial bypass for patients with hemorrhagic moyamoya disease: results of the Japan Adult Moyamoya Trial. *Stroke*. May 2014;45(5):1415-21. doi:10.1161/STROKEAHA.113.004386

3. Kim JA, Yoon S, Kim LY, Kim DS. Towards Actualizing the Value Potential of Korea Health Insurance Review and Assessment (HIRA) Data as a Resource for Health Research: Strengths, Limitations, Applications, and Strategies for Optimal Use of HIRA Data. *J Korean Med Sci*. May 2017;32(5):718-728. doi:10.3346/jkms.2017.32.5.718

4. Yoshida Y, Yoshimoto T, Shirane R, Sakurai Y. Clinical course, surgical management, and long-term outcome of moyamoya patients with rebleeding after an episode of intracerebral hemorrhage: An extensive follow-Up study. *Stroke*. Nov 1999;30(11):2272-6. doi:10.1161/01.str.30.11.2272

5. Morioka M, Hamada J, Todaka T, Yano S, Kai Y, Ushio Y. High-risk age for rebleeding in patients with hemorrhagic moyamoya disease: long-term follow-up study. *Neurosurgery*. May 2003;52(5):1049-54; discussion 1054-5.

6. Kang S, Liu X, Zhang D, et al. Natural Course of Moyamoya Disease in Patients With Prior

© 2024 Lim YC et al. *JAMA Network Open*.

Hemorrhagic Stroke. *Stroke*. May 2019;50(5):1060-1066. doi:10.1161/STROKEAHA.118.022771

7. Zhao M, Deng X, Gao F, et al. Ischemic Stroke in Young Adults with Moyamoya Disease: Prognostic Factors for Stroke Recurrence and Functional Outcome after Revascularization. *World Neurosurg*. Jul 2017;103:161-167. doi:10.1016/j.wneu.2017.03.146

8. Lim YC, Lee E, Song J. Depression or Anxiety According to Management Modalities in Patients With Unruptured Intracranial Aneurysms. *Stroke*. Dec 2022;53(12):3662-3670. doi:10.1161/STROKEAHA.122.040330

9. Nguyen VN, Motiwala M, Elarjani T, et al. Direct, Indirect, and Combined Extracranial-to-Intracranial Bypass for Adult Moyamoya Disease: An Updated Systematic Review and Meta-Analysis. *Stroke*. Dec 2022;53(12):3572-3582. doi:10.1161/STROKEAHA.122.039584

10. Kim T, Oh CW, Bang JS, Kim JE, Cho WS. Moyamoya Disease: Treatment and Outcomes. *J Stroke*. Jan 2016;18(1):21-30. doi:10.5853/jos.2015.01739

11. Scott RM, Smith ER. Moyamoya disease and moyamoya syndrome. *N Engl J Med*. Mar 19 2009;360(12):1226-37. doi:10.1056/NEJMra0804622

12. Cho WS, Kim JE, Kim CH, et al. Long-term outcomes after combined revascularization surgery in adult moyamoya disease. *Stroke*. Oct 2014;45(10):3025-31. doi:10.1161/STROKEAHA.114.005624

13. Kim T, Oh CW, Kwon OK, et al. Stroke prevention by direct revascularization for patients with adult-onset moyamoya disease presenting with ischemia. *J Neurosurg*. Jun 2016;124(6):1788-

© 2024 Lim YC et al. *JAMA Network Open*.

93. doi:10.3171/2015.6.JNS151105

14. Bang JS, Kwon OK, Kim JE, et al. Quantitative angiographic comparison with the OSIRIS program between the direct and indirect revascularization modalities in adult moyamoya disease. *Neurosurgery*. Mar 2012;70(3):625-32; discussion 632-3. doi:10.1227/NEU.0b013e3182333c47

15. Arias EJ, Dunn GP, Washington CW, et al. Surgical Revascularization in North American Adults with Moyamoya Phenomenon: Long-Term Angiographic Follow-up. *J Stroke Cerebrovasc Dis*. Jul 2015;24(7):1597-608. doi:10.1016/j.jstrokecerebrovasdis.2015.03.053

16. Kuroda S, Houkin K, Ishikawa T, Nakayama N, Iwasaki Y. Novel bypass surgery for moyamoya disease using pericranial flap: its impacts on cerebral hemodynamics and long-term outcome. *Neurosurgery*. Jun 2010;66(6):1093-101; discussion 1101. doi:10.1227/01.NEU.0000369606.00861.91

17. Nielsen TH, Abhinav K, Sussman ES, et al. Direct versus indirect bypass procedure for the treatment of ischemic moyamoya disease: results of an individualized selection strategy. *J Neurosurg*. Jun 12 2020;134(5):1578-1589. doi:10.3171/2020.3.JNS192847

18. Kuroda S, Houkin K. Moyamoya disease: current concepts and future perspectives. *Lancet Neurol*. Nov 2008;7(11):1056-66. doi:10.1016/S1474-4422(08)70240-0

19. Deng X, Gao F, Zhang D, et al. Direct versus indirect bypasses for adult ischemic-type moyamoya disease: a propensity score-matched analysis. *J Neurosurg*. Jun 2018;128(6):1785-1791. doi:10.3171/2017.2.JNS162405

© 2024 Lim YC et al. *JAMA Network Open*.

20. Abla AA, Gandhoke G, Clark JC, et al. Surgical outcomes for moyamoya angiopathy at barrow neurological institute with comparison of adult indirect encephaloduroarteriosynangiosis bypass, adult direct superficial temporal artery-to-middle cerebral artery bypass, and pediatric bypass: 154 revascularization surgeries in 140 affected hemispheres. *Neurosurgery*. Sep 2013;73(3):430-9. doi:10.1227/NEU.0000000000000017

21. Macyszyn L, Attiah M, Ma TS, et al. Direct versus indirect revascularization procedures for moyamoya disease: a comparative effectiveness study. *J Neurosurg*. May 2017;126(5):1523-1529. doi:10.3171/2015.8.JNS15504

22. Jeon JP, Kim JE, Cho WS, Bang JS, Son YJ, Oh CW. Meta-analysis of the surgical outcomes of symptomatic moyamoya disease in adults. *J Neurosurg*. Mar 2018;128(3):793-799. doi:10.3171/2016.11.JNS161688

23. Sun H, Wilson C, Ozpinar A, et al. Perioperative Complications and Long-Term Outcomes After Bypasses in Adults with Moyamoya Disease: A Systematic Review and Meta-Analysis. *World Neurosurg*. Aug 2016;92:179-188. doi:10.1016/j.wneu.2016.04.083

24. Yu J, Shi L, Guo Y, Xu B, Xu K. Progress on Complications of Direct Bypass for Moyamoya Disease. *Int J Med Sci*. 2016;13(8):578-87. doi:10.7150/ijms.15390

© 2024 Lim YC et al. *JAMA Network Open*.
