# Supplementary material for: Outcomes of Bypass Surgery in Adult Moyamoya Disease by Onset Type
Source: JAMA Netw Open. 2024 Jun 6;7(6):e2415102. doi: 10.1001/jamanetworkopen.2024.15102 (PMC11157360; doi:10.1001/jamanetworkopen.2024.15102)
Supplement: Supplement 2. — Data Sharing Statement [file jamanetwopen-e2415102-s002.pdf]

Data Sharing Statement

Lim. Outcomes of Bypass Surgery in Adult Moyamoya Disease by Onset Type. *JAMA Netw Open*. Published June 05, 2024. doi:10.1001/jamanetworkopen.2024.15102

Data
Data available: No

Additional Information
Explanation for why data not available: All data is available from the database of Korea national health insurance sharing service (https://nhiss.nhis.or.kr). KNHISS does not allow researchers to provide data to other sites personally. Therefore, the authors do not have the right to provide materials to another person or institution. In order to access the original data of this paper, you can follow the KNHISS guidelines and promise to follow the research ethics through the website, and then provide a certain fee and request the raw data. This process requires IRB approval. Everyone can apply for the data at the HIRA-NPS web site (http://nhiss.nhis.or.kr/, tel: +82-33-736-2430, 2431). These processes are intended to get consent from all researchers for the compliance of ethical guidelines not to impede the data sharing. The authors did not have special access privileges to these data sets.
